# Mortar Synthesis: A Mechanochemical Approach to Metal Aerogel Production

**DOI:** 10.1021/acs.chemmater.5c01607

**Published:** 2025-10-24

**Authors:** Johannes Kresse, Laura Uhlmann, Annika Christiansen, René Hübner, Alexander Eychmüller

**Affiliations:** a Physical Chemistry, 9169 TU Dresden, Zellescher Weg 19, 01069 Dresden, Germany; b Leibniz-Institut für Polymerforschung Dresden e. V., Hohe Straße 6, 01069 Dresden, Germany; c Institute of Ion Beam Physics and Materials Research, 28414 Helmholtz-Zentrum Dresden-Rossendorf e.g., 01328 Dresden, Germany

## Abstract

Metal aerogels are versatile materials that bind the nanoparticulate properties of their building blocks in a self-supporting macroscopic body. Their large specific surface area (SSA), conductivity, and electrocatalytic activity make them highly attractive for a wide range of applications. High production rates are, therefore, equally important. To this end, we report a unique approach to synthesize metal aerogels in a mortar, operating at the highest metal precursor concentrations currently reported. The metal precursor and NaBH_4_ are ground together, initiating partial reduction by mechanochemical activation, followed by adding water to complete the reduction. In this way, aerogels are successfully prepared from all nonradioactive metals of groups 8–11, for the first time from the main group elements In, Sb, Pb, Bi, and bimetals thereof. Particularly, the Fe aerogel morphology can be altered between random interconnected networks and nanochain bundles under the influence of an external magnetic field. Depending on the element, SSAs between 2 and 70 m^2^/g and ligament sizes of 2–140 nm were achieved. On top of this great versatility, gelation times are often unprecedentedly fast, i.e., almost instantaneous, and easily achieve remarkably high state-of-the-art production rates of at least 10 mmol per batch, often with minimal trade-off in ligament size and SSA.

## Graphical abstract

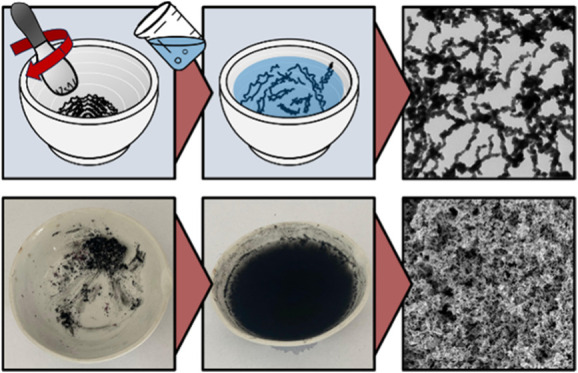

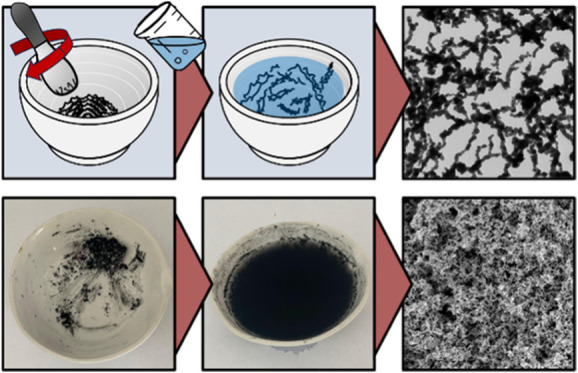

## Introduction

Over the past decade, metal aerogels have transpired into one of the most promising classes of emerging materials.^1^ They are characterized by their intrinsic nanoproperties, namely, a high specific surface area (SSA), small ligament size (strand diameter), and extremely low density. Equally important is the incorporation of the nanoproperties into a macroscopic self-supporting body, allowing fast and easy processing of metal aerogels.^2,3^ Accompanied by high activity, selectivity, and conductivity, such materials are highly attractive for electrocatalysis in fuel cells, sustainable energy cycles, and more.^4–6^ This is also reflected in the multitude of different building blocks (spheres, cuboids, sheets, rods, ...),^7–12^ elements (noble metals, magnetic metals, metalloids, ...),^13–17^ element distributions (segregated, alloy, core–shell, ...),^7,13,18^ and structural fine-tunings (surface composition,^19–22^ d-band center,^23–26^ spin,^27,28^ defects,^29–31^ doping,^32–35^ lattice facet control,^36–38^ ...), that have been reported since the popularization of metal aerogel synthesis by Bigall et al. in 2009.^39^

Beyond the conventional sol–gel-based approach, several adjacent fields, such as dealloying^40^ or vapor deposition,^41^ can also yield aerogel-like structures. A special case is mechanochemistry.^42^ It uses mechanical energy to induce a chemical transformation, typically by grinding, milling, or shearing. While mechanochemical synthesis is well established for NPs,^43–45^ its application to metal aerogels has so far been only incidental; aerogel-like structures have been observed, but they were neither recognized as such nor the primary focus of the work. Early studies reported ball milling of base metal salts with Na as the reductant and NaCl as dilutant to prevent combustion.^46–49^ Other possible reductants include Al, B, Cr, and Si.^50^ A later report replaced Na/NaCl with Ca/CaO,^51^ representing the only significant follow-up study to date. Despite the scalability advantage of solid-state over solution-based methods, their numerous limitations likely hindered broader adoption. The synthesis requires large amounts of dilutant salt, often 1 to 3.5 times the molar quantity of the metal precursor. The benefit of a “dry” product is negated by the need for purification by solvent washing. Omitting the dilutant leads to combustion and the formation of dense, nonporous aggregates. Moreover, complete reduction of the metal precursor may require several hours of milling, during which prolonged mechanical stress coarsens the aerogel structure.^47^ Lastly, the target metal alloys with the reductant, necessitating postsynthetic removal when undesired.^50,51^ Even less researched is liquid-assisted mechanochemistry, in which a tiny amount of liquid is added to accelerate the mechanical activation without causing complete solvation.^52^

When comparing the different sol–gel based fabrication strategies,^14^ NaBH_4_-triggered reduction shows the overall best performance and versatility. This includes fast gelation, low costs, many available elements, and good structure control. Only in terms of yield and purity, the diluted batch process and uncontrolled surface chemistry cause limitations. Alternative fabrication strategies can overcome these. For example, freeze–thawing^53^ facilitates gelation by concentrating the nanoparticles (NPs) between the solvent crystals during freezing (−196 °C) and releases the gel upon thawing. This enables higher yields but only at the expense of longer synthesis times, higher costs, and even less structure control. Similar considerations can be made for heating,^54^ salting out,^55^ oxidation,^39^ and any other gelation method.^14^ However, not all gelation strategies are versatile; some are specific and either enable synthesis (many galvanic replacement reactions) or optimize certain properties in compromise of most others. One example is the dopamine-triggered gelation of Au NP,^56^ which currently offers the smallest possible ligament size and excellent structure control in exchange for a long production time and low yield.

In particular, base metal aerogels (Fe, Ni, Co, Cu) tend to be studied in isolation, leading to various possible gelation strategies despite the few publications. These reports include hydrazine^57–60^ or NaBH_4_-triggered reduction,^35,61–64^ deoxidation,^65^ high-temperature reduction (H_2_ atmosphere),^66^ vacuum reduction (Schlenk line),^67^ freeze-casting,^68^ and external magnetic fields (Helmholtz coil,^57,58^ solenoid,^69,70^ static,^60,71,72^ spinning magnet^59^). All these methods are not necessarily specific, but apart from a few reports,^35,62,67^ focus only on a single metal or bimetallic composition and thus do not provide a clear connection to systems of other elements. Although the alternative process of nano smelting is more universal, it is, in turn, limited to carbothermally reducible metals. This excludes noble metals.^73,74^ More emphasis should be placed on genuinely specialized strategies, such as magnetic fields or versatile methods that apply equally to base and noble metals. Although noble metal gelations often exhibit such versatility, pure base metals are commonly still omitted from these syntheses.^17,64,75–77^ This could be due to their poor chemical stability in oxidative environments,^78–81^ which worsens as the ligament size becomes smaller.^82^ In turn, the abundance of base metals in the Earth’s crust is several orders of magnitude higher, enabling cost-efficient production, which offsets their lower activity and stability.^83^ This also means a lower environmental impact due to easier extraction. Examples are Fe catalysts for the Fischer–Tropsch process^84^ and pharmaceuticals^85^ or Ni for steam reforming.^86,87^ An efficient catalyst design should consider all these factors simultaneously.

Regardless of the gelation strategy, the limited production rate is currently the biggest drawback of metal aerogel synthesis. Much more so than the need for supercritical or freeze-drying, as industrial autoclaves and freeze-dryers can operate in the kiloliter range.^88,89^ Furthermore, alginate gels have already been dried continuously.^90^ The simplest way to up-scale the production is to increase the overall batch size.^91,92^ However, this only works to a certain extent, as the relative dimensions of the reaction vessel and precipitation distance should be maintained as much as possible. Therefore, most works focus on shortening the gelation time to a few hours at most. This includes enhanced Brownian motion at higher temperatures^93–99^ (including reflux^100^ and hydro/solvothermal conditions^101,102^ ), increased NP collisions by agitation (stirring rods,^77,103^ sonication,^104^ shanking,^96^ or centrifugation^101,105,106^ ), weaker NP stabilization through salting-out,^55,107–109^ seed supported growth,^110^ and higher nucleation rates due to increased metal precursor concentrations.^75,103,111–113^ However, except for many high-concentration methods, this also increases chemical and energy consumption. Of particular note is microwave heating, which produces gels in less than a minute.^114^ This acceleration correlates with the microwave power but can also lead to a broadening of the ligaments. The low amount of metal precursor (6.5 times less than Liu’s original one-step gelation^76,115^ ), further negates the time gain as more manual labor is required. One of the more efficient up-scalings to date was achieved by Georgi et al. by changing the solvent from water to ethanol and concentrating the metal precursor by a factor of 50–100.^64^ This increases the nucleation rate and changes the dielectric constant from 80 (H_2_O) to 25 (EtOH), which thins the solvent shells and fastens gelation.^116,117^ Recently, even a continuous gelation of 2D metal gels was reported. However, due to low laminar flow rates of 3 mL/h, it is impractical for 3D gels.^118^ Similarly, metal aerogel printing is hindered by long printing and aging times.^119^ Since the gelation kinetics are partially intertwined with the batch volume, they cannot be clearly disentangled. Ideally, a simultaneous improvement of all three factors—gelation kinetics, metal concentration, and batch volume—would have the most positive effect on up-scaling the synthesis.

A detailed comparison of the gelation time, ligament size, SSA (inconsistently reported due to missing data or lack of supercritical/freeze-drying), and yield of different metals, with particular emphasis on Fe and Pd, is shown in Figure 1. The corresponding reaction conditions are listed in Table S1. Compared are the respective peak values of each reference, which may not necessarily originate from the same gel. Mean gelation times typically range from 10 min to 10 h, while the SSAs do not show a clear average. In contrast, the ligament size and yield each exhibit two average ranges between 2 and 6 nm and 50–150 nm, as well as 0.08–0.4 mmol and 3–10 mmol, corresponding to low and high metal precursor concentrations, respectively. This division roughly reflects the separation in noble and base metals, with the latter typically employing higher precursor amounts and metal salt concentrations. Overall, an increasing metal salt concentration tends to shorten the gelation time, broaden ligament size, decrease SSA, and increase yield, largely independent of the gelation strategy. This highlights the metal precursor concentration as a superordinate key parameter. Likewise, faster gelation often correlates with larger ligaments. However, these trends are not universal, as numerous factors such as ligand, salt, heat, etc., also affect gelation and possibly counteract their effect. For example, templating (u) retains smaller ligaments than regular one-step gelation (o) despite shorter synthesis times, whereas high-temperature polyol synthesis (r) results in larger ligaments despite longer gelation. The existence of many similar cases (ó, ñ, ú; k, j, h; ...) emphasizes the decisive role of the reaction condition besides the metal salt concentration. While mechanical disturbance (h), microwaves (s), and magnetic fields (w) enable the fastest gelations, the smallest ligaments are obtained by oriented attachment through π-π stacking (p, q, g) or polar interactions (i) as well as polyol synthesis (v), and the highest yields from concentrated approaches with large precursor amounts supported by magnetic fields, disturbance, or polyol synthesis. Nevertheless, no single approach achieves peak performance in multiple of the four properties simultaneously, as trade-offs in the others always occur. Moving from ligand- and additive-free approaches, which are economically and ecologically favorable but limited in tunability, toward additive-assisted strategies provides a more balanced performance. Notable examples are radiolytic reduction combined with hexagonal mesophase templating (u; not supercritically dried) and two-step gelation in which destabilization is triggered by salting-out with NH_4_Cl (ó). Compared to the literature, the mortar synthesis for Pd (red stars) simultaneously achieves the shortest gelation time and highest metal salt concentration reported to date, while maintaining significantly small ligament sizes and high SSAs. Furthermore, even though the trade-offs in the latter two properties are not entirely eliminated, they are substantially minimized compared to all previous reports, while also returning to the simplicity of a ligand- and additive-free approach. For Fe (red hollow stars), the mortar synthesis performs slightly worse than Pd in most properties, but this is element-specific, and compared with base metals alone, it is equally outstanding. Important to note, the up-scaling of the metal salt concentration and metal precursor amount was split onto Pd and Fe, respectively; combining both in one system could further increase the scalability by factors of several hundred or more.

**Figure 1:**
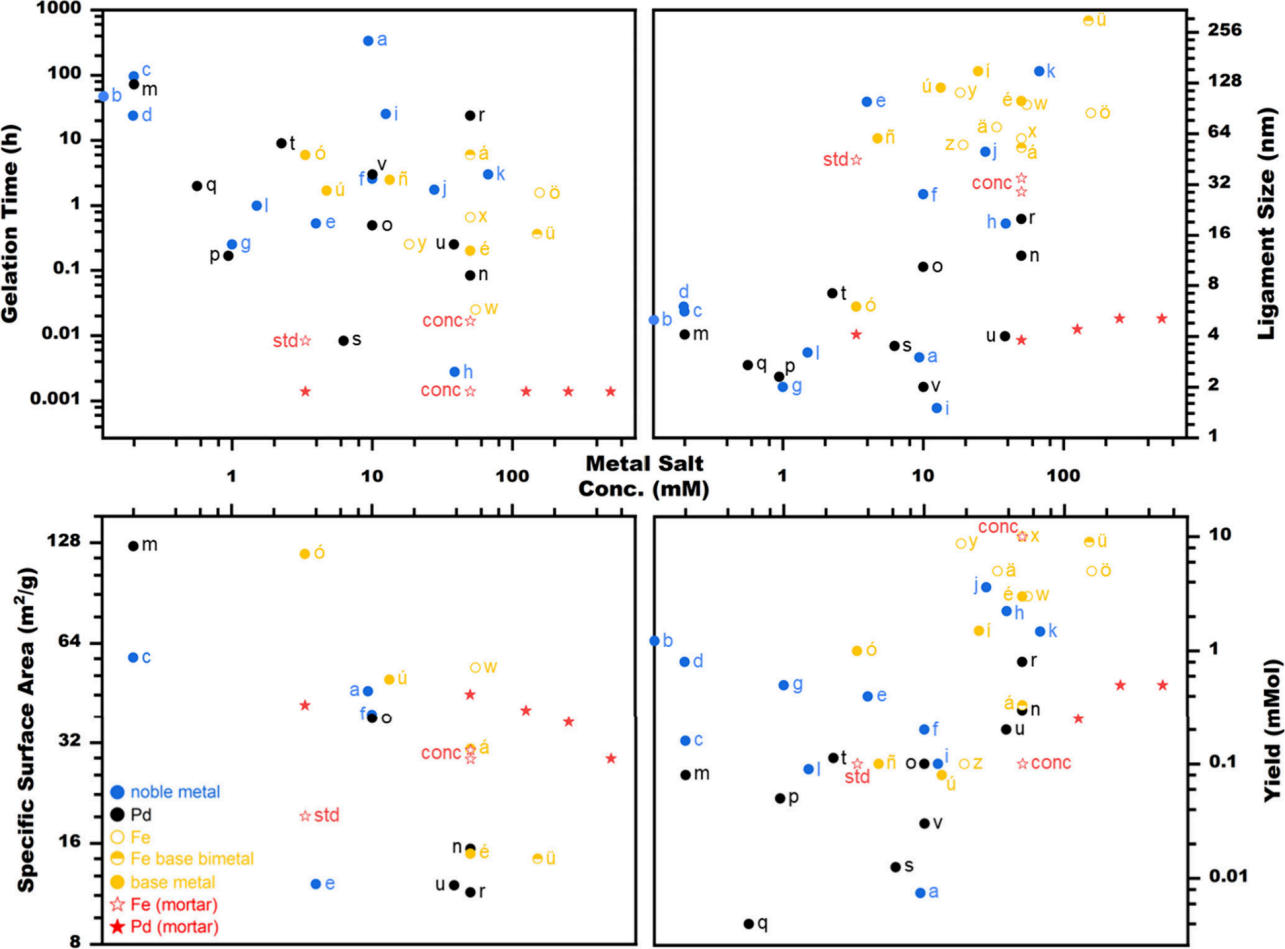
Comparison of the key properties gelation time, ligament size, SSA, and yield for various noble metal (blue), Pd (black), and Fe/base metal (yellow) aerogels reported in the literature with Pd (red star) and Fe (red hollow star) aerogels obtained via mortar synthesis. For Fe, “std” and “conc” denote standard and concentrated mortar conditions, respectively. The previous reports generally show that an increasing metal salt concentration decreases gelation time and SSA while increasing ligament size and yield. Shorter gelation often coincides with larger ligaments, though not universally. Compared to the literature, the mortar synthesis achieves the fastest gelation and highest metal salt concentration to date, with only minor trade-offs in the other key properties and without using ligands or additives. Related references: (a) Bigall et al.,^39^ (b) Qian et al.,^110^ (c) Henning et al.,^120^ (d) Fikry et al.,^91^ (e) Feng et al.,^107^ (f) Tang et al.,^96^ (g) Liu et al.,^105^ (h) Yan et al.,^103^ (i) Zhao et al.,^101^ (j) Qian et al.,^108^ (k) Peng et al.,^98^ (l) Zhang et al.,^118^ (m) Liu et al.,^76^ (n) Burpo et al.,^112^ (o) Georgi et al.,^64^ (p) Zheng et al.,^106^ (q) Wang et al.,^109^ (r) Wang et al.,^102^ (s) Zhao et al.,^114^ (t) Martínez-Lázaro et al.,^121^ (u) Ksar et al.,^122^ (v) Wang et al.,^99^ (w) Calabro et al.,^69^ (x) Li et al.,^113^ (y) Krajewski et al.,^123^ (z) Lin et al.,^124^ (ä)Li et al.,^97^ (ö) Bian et al.,^100^ (ü) Liang et al.,^57^ (á) Yan et al.,^62^ (é) Zou et al.,^59^ (í) You et al.,^71^ (ó) Huang et al.,^61^ (ú) Zhao et al.,^72^ (ñ) Tang et al.^68^

To address the lack of a versatile, high-yield metal aerogel production method, we developed a synthesis strategy that enables near-instantaneous gelation for many noble, base, metalloid, and multimetallic systems while preserving the simplicity of the NaBH_4_-triggered reduction. In a mortar, we thoroughly grind NaBH_4_ and the metal salt to trigger an initial reduction, which is completed by adding water. In the case of magnetic aerogels (Fe, Co, Ni), the gelation kinetics are further improved by applying an external magnetic field using a strong permanent magnet. For Fe, in particular, this also leads to controllable morphological changes. Hence, it became our model system to investigate the individual synthesis parameters and their influence on the resulting aerogel morphology and ligament properties. These parameters include the metal concentration, solvent, additives, milling, and more. Furthermore, we characterized the aerogels regarding crystallinity, oxidation states, and porosity.

## Experimental Section

### Chemicals

FeCl_3_ (97%, Fluka), CoCl_2_·6 H_2_O (98%, abcr), NiCl_2_·6 H_2_O (99.9%, Sigma-Aldrich), CuCl_2_·2 H_2_O (reagent grade, Sigma-Aldrich), PdCl_2_ (99.999%, Sigma-Aldrich), H_2_PtCl_6_·x H_2_O (99.9%, Acros Organics), HAuCl_4_·3 H_2_O (99.99%, abcr), InCl_3_ (99.999%, Sigma-Aldrich), RuCl_3_ (99.9%, Sigma-Aldrich), RhCl_3_ (99.9%, Alfa Aesar), K_2_IrCl_6_ (99.99%, Sigma-Aldrich), OsCl_3_·x H_2_O (99.9%, Sigma-Aldrich), AgCl (99.5%, Sigma-Aldrich), SbCl_3_ (99.95% Sigma-Aldrich), PbCl_2_ (98%, Sigma-Aldrich), BiCl_3_ (98%, Chempur), NaBH_4_ (granular, 99.99% Sigma-Aldrich), Na_3_Cit (p.a., Merck), Acetone (p.a., Sigma-Aldrich), and CO_2_ (99.8%, Air Liquide) were used as received. Ethanol (denatured with 1% petroleum ether, Berkel AHK) was dried over a molecular sieve 3 Å (0.3 nm, type 564, pearl shape, Carl Roth). Milli-Q water (0.056 μS cm^−1^) was provided by a coupled system of RiOs 8 and Milli-Q Academic from Millipore.

### Mortar Gelation of Metal Aerogels

The synthesis procedure is illustrated in Figure 2 and Video S1. Metal precursor and NaBH_4_ were weighed in at a ratio of electron vacancies to hydride ions of 1 to 8 (*n*_*Me* ^*i*+^_:*n*_*H* ^–^_). Afterward, the 8 equiv (Eq) of NaBH_4_ were ground to a fine powder in a porcelain mortar. Grinding should be short and immediately before the reaction to avoid the early attraction of water and lumping. Subsequently, 0.1 mmol of metal precursor was added, and the mixture ground again, whereby a black discoloration accompanied by smoke development indicated the beginning of the reduction. At this point, 30 mL DI water was added in one swift motion. Immediately after the precipitation of the hydrogel (latest after 30 min), it was washed three times in succession by removing excess solvent and adding fresh DI water. Any ungelled supernatant was discarded. Water was then replaced five times with acetone using the same procedure. Finally, the solvogel was supercritically dried in an autoclave with CO_2_ at 37 °C and 85 bar. The synthesis parameters stated above are standard conditions; deviations are explicitly mentioned in the discussion. Concentrated (conc.) mortar conditions: 0.1 mmol metal precursor, 20 Eq NaBH_4_, plus 2 mL H_2_O and the 100-fold up-scaled variation (10 mmol metal precursor, 20 Eq NaBH_4_, plus 200 mL H_2_O) were also used frequently.

**Figure 2:**
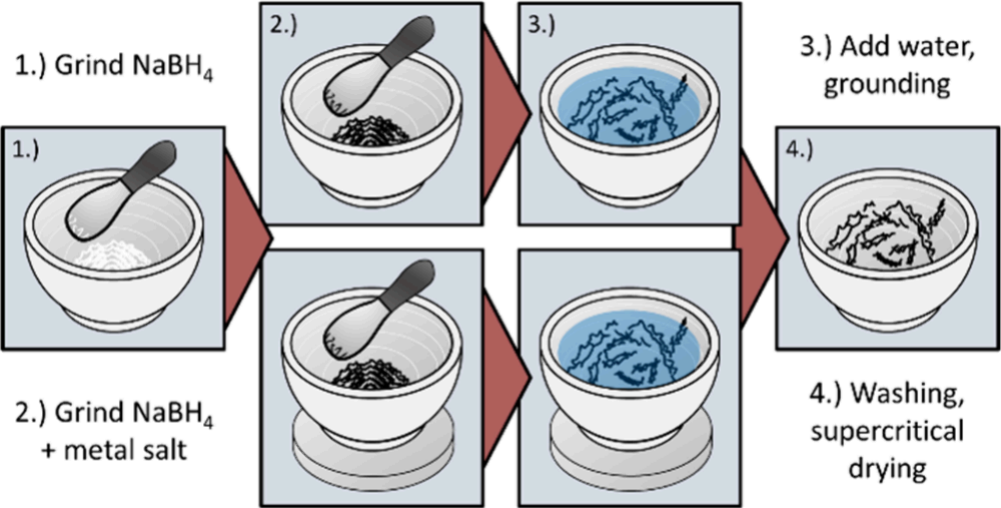
Synthesis scheme of the aerogel production using the mortar approach, without (top) and with (bottom) magnet.

A strong NdFeB magnet can be placed under the mortar to accelerate the gelation time of magnetic gels. Depending on the desired morphology of Fe—nanochain bundles, sponge-like, or hybrid—the magnet was placed before, ≳3 s delayed, or immediately (≈1 s) after reduction, respectively.

### Instruments and Characterization

The external magnetic field was generated by cylindrical NdFeB magnets (1.33–1.36 T) with a diameter of 100 mm and heights of 10, 20, and 30 mm, corresponding to an adhesive force of 2550, 3190, and 3630 N. Supercritical CO_2_-drying was performed in an autoclave of the model 13200J0AB from Spi Supplies. For structure analysis, the gels were examined using a Zeiss Libra 120 transmission electron microscope (TEM) equipped with a LaB_6_ cathode and operated at an accelerating voltage of 120 kV. Likewise, an FESEM SU8020 scanning electron microscope (SEM) from Hitachi was used (3 kV, 7 μA). Phase analysis was performed via a Bruker D2 Phaser powder X-ray diffractometer (Cu K 1.5406 Å). To measure the specific surface area, total pore volume (TPV), and pore size distribution of the aerogels, N_2_ physisorption was performed at standard temperature (77 K) and pressure (1 atm) at p/p0 = 0.98 on a model Nova 3000e from Quantachrome. The Brunauer–Emmett–Teller theory was used to calculate the respective parameters, except for the pore size distribution, which was calculated according to the Barrett–Joyner–Halenda theory. To analyze the element composition at the nanoscale, high-angle annular dark-field (HAADF) scanning transmission electron microscopy (STEM) and spectrum imaging analysis based on energy-dispersive X-ray spectroscopy (EDX) were conducted on a Talos F200X transmission electron microscope (FEI) operated at 200 kV and equipped with the ChemiSTEM technology for fast EDX acquisition, including four windowless silicon drift detectors (Super-X) and a high-brightness X-FEG electron source. X-ray photoelectron spectroscopy (XPS) measurements were performed using a Kratos AXIS Ultra DLD to evaluate the element compositions. Grammatical corrections were made with DeepL and Grammarly.

## Results and Discussion

### Gel Formation in a Mortar

The gelation process of Fe is shown in Figure 3. Unlike traditional one-step gelations it undergoes a two-part reduction, first in the solid and then liquid phase. When grinding the metal precursor and NaBH_4_, i.e., mechanochemical activation, the applied mechanical force is sufficient to initiate a partial reduction, which continues as a self-sustaining exothermic reaction. Visually, this is observed as the discoloration of both reactants into a gray/black solid. Its consistency changes from powder to foam as the hygroscopicity of the metal salt increases. This shifts the partial reduction from a solid-state-like reaction closer toward a concentrated one-step gelation. As a result, the solvation by air humidity increases, making the partial reduction more complete. An increasing relative humidity likely has a similar effect which mutually intensifies with the hygroscopicity. Due to the presence of crystal, adsorbed, and in situ generated water, however, this was not further investigated. The discoloration is further accompanied by a pungent gas. pH tests (≈6) and the use of metal chlorides suggest the formation of HCl. TEM images of the intermediate (Figure 3b) reveal three different morphologies: wrinkled sheets, aggregates of NPs (3.8 ± 1.6 nm), and nanosticks (⌀ 5.5 ± 1.2 nm), all featuring first gel characteristics as no ligands are applied in the synthesis. Thereof NPs show the highest abundancy. However, the solid-state reduction by mechanochemical activation is not a complete reaction; individual chunks of NaBH_4_ and metal precursor can be recognized in the discolored solid. It is, therefore, conceivable that some metal salt particles have only been reduced on the surface while their core remains ionic. Following this assumption, the partial reduction ends when most of the precursor has passed into this state. By adding water, such unreacted reactants are immediately dissolved and consequently also reduced. Gelation can finish just as quickly, but also take longer, depending on the element and precursor concentration. The division of the reduction into two steps is of great importance. It decreases the effective amount of metal salt participating in each step, allowing for higher initial concentrations than reported in literature. Adding water further compensates for the lack of mixing in the solid phase. It simultaneously dissolves the remaining NaBH_4_ and metal salt while also agitating the solution by convection (pouring water in the mortar) and H_2_ gas generation. In addition water serves as dispersing medium in which the solid intermediates formed during the partial reduction are swollen and torn into smaller pieces that serve as growth centers for gelation. H_2_ is of particular importance; its numerous generation (1.5–6.5 mg), adsorption on the surface, and intercalation into the network cause the gel fragments to float atop the water for several minutes due to the buoyancy of the gas bubbles. This is closely related to bubble templating,^125–127^ which uses in situ generated gas as a template to prepare porous structures. The mortar approach utilizes the same templating effect but complements it with solution agitation, spatial separation, and tearing of the solid formed by the partial reduction. The higher H_2_ generation compared to the literature further promises more efficient templating. Due to the absence of ligands a temporary stable sol forms, which is mixed with the fragmented intermediates and settles as soon as the convection-induced movements and the H_2_ bubbles subside. After adding water, the solution also splits into two fractions. One with larger gel fragments, which settle within minutes and make up the majority of the sample, and one with smaller gel fragments and NPs, which only settle after hours. The latter is comparable to solution-based one-step gelations and occurs due to the dissolution of unreduced metal salt chunks, cores, and NaBH_4_. Likewise, it explains the long gelation time of the second fraction. The metal salt concentration determines the ratio of both fractions, and if high enough, only the first fraction forms due to the high supersaturation (Video S1). TEM micrographs of the finished Fe gels (Figure 3d) show the typical morphology of three-dimensional, randomly interconnected nanochains built from the intermediately formed NPs. Compared to the literature, they exhibit a broader size and shape distribution. Even neighboring NPs can differ drastically. These differences are already present for the NP and nanostick intermediates and passed on to the gel network. The smallest ligaments might as well originate from the one-step-like fraction. All this is a consequence of the rapid reduction and gelation initiated by the mortar synthesis, making the NPs more inhomogeneous, shriveled, and not necessarily spherical. In addition, wrinkled sheet and needle-like substructures form on the surface of the gel. This could be due to surface deposition of the sheet intermediate, further oxidation of the gel, or building blocks. Higher magnification images (Figure S2) also reveal thin films that appear to be passivation layers covering each strand. This is later validated by STEM-EDX. Strikingly, the size difference between the mortar (5 cm diameter) and the gel (10–50 nm ligament size) is several orders of magnitude. Nevertheless, it is possible to synthesize nanostructured networks, probably due to a complex interplay of all the above-mentioned factors.

**Figure 3:**
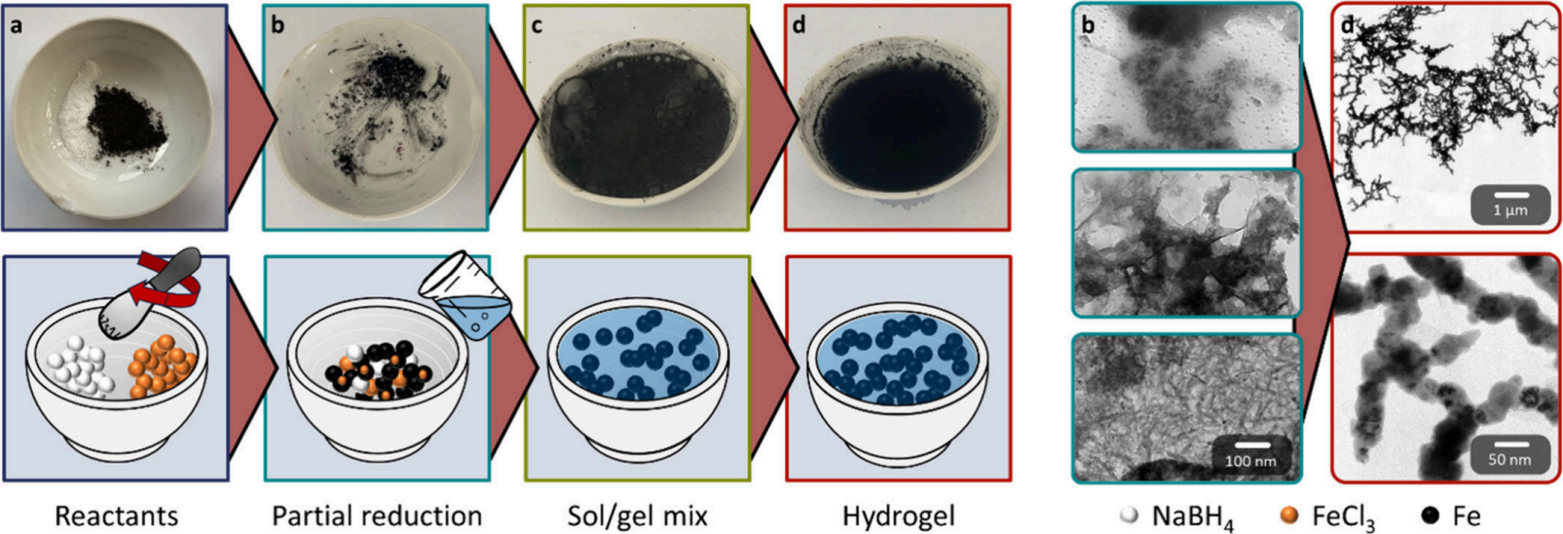
Photo series and proposed gelation mechanism of the mortar synthesis for the example of Fe (left). At first, NaBH_4_ is ground to a fine powder (a) before adding FeCl_3_ and grinding both powders together again to initiate a partial solid state reduction (b). Adding water immediately dissolves the remaining precursor and completes the reduction. At the same time, intensive H_2_ gas evolution and water convection tear apart the already reduced Fe gel intermediates, which act as growth centers of the gelation (c). Depending on the element and concentration, the gelation completes within seconds to minutes (d), perhaps poorly visible as the gel turns the solution opaque. The corresponding TEM micrographs show the three different reaction intermediates before adding water (b) and the final gel (d) after complete grounding (right).

Another photo series in combination with XRD compares the Fe gels from the mortar synthesis, Liu’s original one-step gelation, and a conc. one-step gelation (13-fold metal salt concentration) also based on Liu’s synthesis (Figure S1).^76^ The latter uses equal amounts of chemicals as the mortar approach. While the original one-step gelation forms an orange-brown precipitate, which is a mix of iron oxides (Fe_2_O_3_, Fe_2_O_4_) and iron oxide-hydroxide (Lepidocrocite), the other methods yield dark gray gels. However, only the mortar synthesis has a dominant Fe phase. In the conc. one-step gelation, it is very faint and the same previously mentioned oxides occur. In addition, a broad hump at low angles indicates the presence of an amorphous phase, presumably a mix of Fe, iron oxides, and iron oxide-hydroxides. The same species could also account for the sheet and needle structures. Regardless of the Fe content, the conc. one-step gelation is, in fact, faster than the standard mortar synthesis. Therefore, it leads to larger ligaments of 70 ± 17 nm compared to 40 ± 13 nm (Figure S2). This highlights the advantages of the mortar approach. The partial reduction lowers the effective amount of metal salt reduced per step, which in turn also reduces the ionic strength. In addition, agitation by H_2_ evolution is gentler than mechanical stirring. All these factors slow down the gelation and decrease the ligament size. The original and conc. one-step gelations are also more susceptible to oxidation because the dissolved metal ions and initial NPs are more spatially separated than the only partially solvated metal salts and NPs in the mortar.

### Fe Aerogels

The ligament size is a key property of metal aerogels, essentially determining their porosity. Accordingly, the discussion places much emphasis on it. Figure 4A and Figure S3 summarize various subtle tendencies in the ligament size between 31 and 46 nm, depending on the synthesis conditions. Increasing the residence time in H_2_O (30 s, 2 min, 30 min), H_2_O volume (2 mL, 10 mL, 30 mL), grinding time (10 s, 30 s, 60 s), and NaBH_4_ amount (8 Eq, 14 Eq, 20 Eq) lead to a slight decrease in ligament size, while increasing the amount of Na_3_Cit (5 mg, 30 mg, 30 mg), EtOH percentage in H_2_O (30%, 50%, 70%), adhesive force of the magnet (2550 N, 3190 N, 3630 N), and pH (2.4, 4, 7) have the opposite effect. These subtle tendencies make the gelation robust to small synthetic changes while also allowing fine-tuning of the ligament size under strong alterations. This is important for later up scaling. However, the ligament size cannot be studied in isolation as synthetic changes also affect other aspects, such as gelation time and oxidation, also summarized in Table S2.

**Figure 4:**
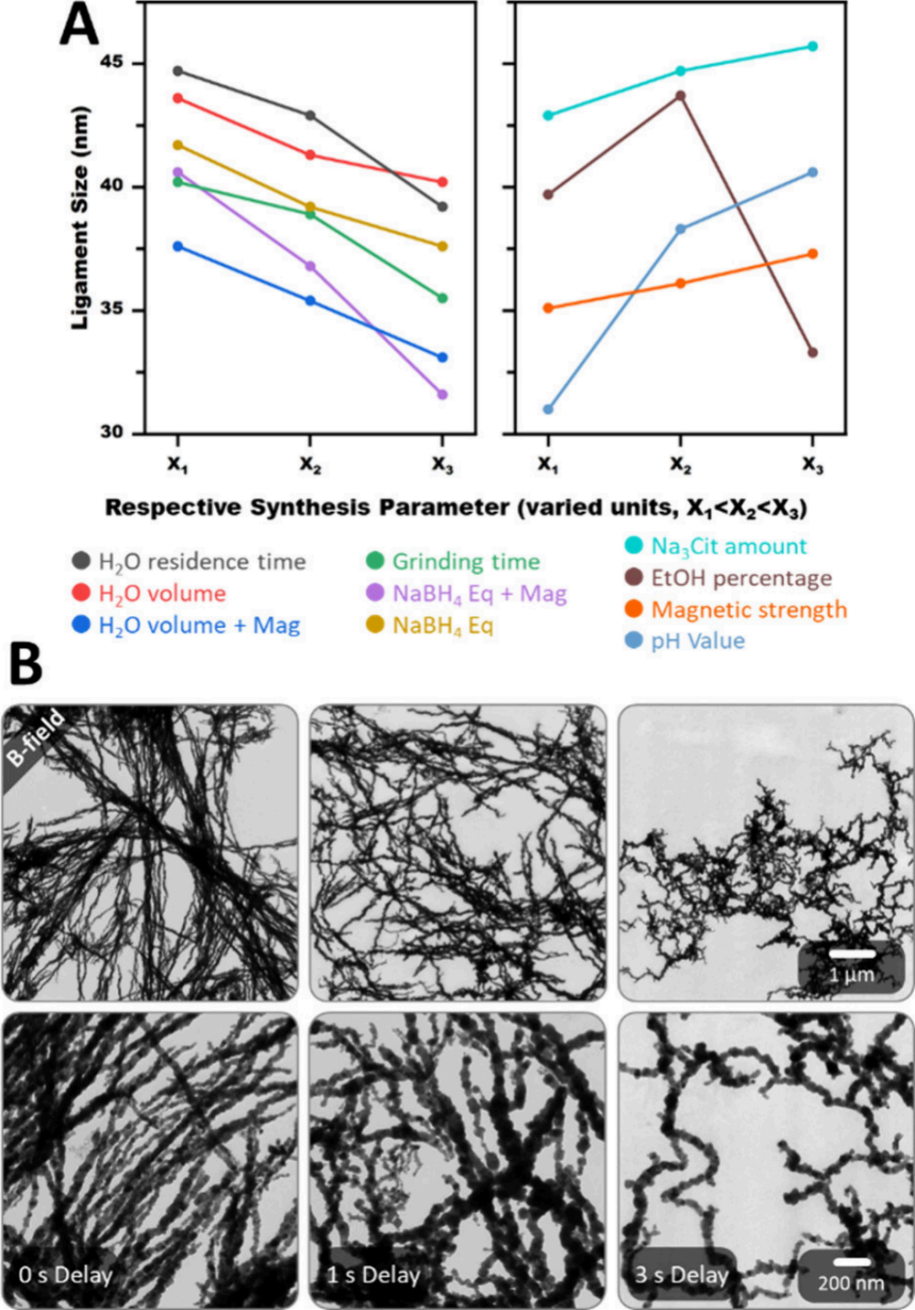
Synthesis parameters reveal several subtle tendencies of the ligament size to decrease with increasing H_2_O residence time (30 s, 2 min, 30 min), H_2_O volume (2 mL, 10 mL, 30 mL), grinding time (10 s, 30 s, 60 s), and NaBH_4_ amount (8 Eq, 14 Eq, 20 Eq), and increase with increasing Na_3_Cit amount (5 mg, 30 mg, 30 mg), EtOH percentage (30%, 50%, 70%), adhesive force of the magnet (2550 N, 3190 N, 3630 N), and pH (2.4, 4, 7) (A). TEM micrographs of Fe aerogels synthesized under standard mortar conditions with different placement times of the magnet: before reduction, 1 s delayed, and 3 s delayed. This results in parallel and double-cone wire bundles, hybrid structures, and random meandering strands, respectively (B).

Grinding in a mortar introduces a set of new parameters: grinding time, grinding force, and grinding order. To investigate these, NaBH_4_ and FeCl_3_ were milled together and sequentially (first NaBH_4_, then NaBH_4_ and FeCl_3_ combined) for 10, 30, or 60 s each before adding water (Figure S4). Only a longer grinding time slightly reduces ligament size by prolonging the partial reduction step, resulting in a higher quantity of intermediate structures. These are thinner than the final gel strands and due to the higher metal consumption undergo less broadening. The other two parameters, grinding force and grinding sequence, leave the ligament size of around 35 nm and gel morphology largely unaffected. This lack of influence may seem inconspicuous, but is of great importance for the standardization of the mortar synthesis, as it excludes the dependence on the operator. The only problem is grinding for too long (few minutes), which attracts more and more air moisture to the precursors, leading to solvation, lumping, and a loss of the two-part reduction.

A prolonged residence time in H_2_O decreases the ligament size by incorporating more of the one-step-like fraction into the gel, but at the same time promotes oxidation. After 30 s, only a passivation layer is formed, while after 2 min, first wrinkled sheets appear, and after 30 min, they cover most of the gel (Figure S5). This correlates with the visually observed sharp drop in H_2_ bubble generation after approximately 1–2 min. As a result, less H_2_ is adsorbed on the NPs/gel, exposing more surface area to oxidation. Therefore, gelation should ideally finish before the H_2_ generation subsides; otherwise, it is best to discard the ungelled supernatant. Another method to suppress oxidation is to lower the pH of H_2_O. This dissolves any oxide that forms, but in part also unselectively removes Fe from the hydrogel. Although this strategy achieves the smallest ligament size of 31 nm, the yield at pH 2.4 and 4 is diminished by 80% and 50%, respectively (Figure S6). Less intrusive are Na_3_Cit additives during grinding. They improve the NP stabilization and protect the surface from oxidation, even at a prolonged residence time in H_2_O of 5 min. The higher ionic strength marginally increases the ligament size but, more importantly, leads to smoother gel strands but also fewer branching points, and less connectivity along the strands (Figures S7, S8). Alternatively, a less oxidizing solvent can be used.^128^ However, counterintuitively, higher EtOH contents promote secondary structure formation. At 50% EtOH, the gel is overgrown by it, and becomes discontinuous at 70% EtOH (Figure S9). In pure EtOH, no gelation occurs, and an orange precipitate, analog to Liu’s one-step gelation in Figure S1, is formed. This is due to the slow dissolution of NaBH_4_ in EtOH, which cannot provide the same instantaneous reduction and mixing as water. As expected, the ligament size increases slightly from 30% to 50% EtOH because of the lower dielectric constant and thus formation of thinner solvent shells. At 70% EtOH, the gel becomes discontinuous and the trend reverses. Furthermore, combining the gelation-accelerating effect of EtOH and gelation-decelerating and surface-protecting effects of Na_3_Cit creates a synergistic behavior. This results in gels with the same meandering network pattern as in H_2_O, no substructure, and ligament sizes 5–10 nm smaller than those without synergistic effect (Figure S10). For 70% and pure EtOH, no synergistic effects were observed.

Higher NaBH_4_ Eq facilitate a faster reduction and formation of smaller initial NPs, which is reflected in a decrease in ligament size (Figure S11). The BH_4_^–^ anion further acts as a ligand and prolongs the NP stabilization. This is particularly important for their separation during grinding, but also extends the gelation time from a few minutes to one or several hours for 8, 14, and 20 Eq, respectively. Ungelled supernatants were discarded after 30 min to exclude the one-step-like fraction of the gel. This corresponds to a 15% and 50% yield loss for 14 and 20 Eq, respectively. Similarly, a larger H_2_O volume decreases the ionic strength and metal concentration, reducing the nucleation rate and extending the gelation time due to fewer NP collisions and thicker solvent shells. Besides smaller ligaments, the effect on the gelation time is even more pronounced. For 2 mL, it is almost immediate, while 10 and 30 mL take 2 and 5 min, respectively (Figure S12). Similar to before, combining a low H_2_O volume and high NaBH_4_ Eq evokes a synergistic effect. The concurrent acceleration and deceleration of the gelation, as well as the increase and decrease in ligament size more or less balance each other out. Under conc. mortar conditions (0.1 mmol metal salt, 20 Eq NaBH_4_, 2 mL H_2_O), this results in gels with even smaller ligaments of 29 ± 9 nm than those of the individual conditions, almost immediate gelation, and hardly any oxidation (Figure S13). The additional decrease in ligament size is likely due to higher in situ H_2_ concentrations and, thus, stronger NP separation. Compared to Liu et al.^76^ and Georgi et al.^64^ (although not shown for Fe), this represents a 200-fold and 2–4-fold increase of the metal precursor concentration, respectively, and a reduction in gelation time from 2 days/3 weeks and 30 min to a few seconds.

Applying an external magnetic field in the form of a strong permanent magnet under the mortar can further reduce the gelation time, e.g., to 2 min for the standard conditions. Simultaneously, it avoids oxidation. Most important, however, is the introduction of an anisotropic field that parallelly aligns the NPs along the magnetic field lines. As a result, fusion occurs predominantly at the tip of the strand and is suppressed at the side, forming parallel wire bundles of up to several micrometers in length. When multiple NPs fuse at once, they create a branching point. Although such assemblies have been reported before (see Introduction), they suffer from large ligament sizes of 150 nm and above, hydrazine as a highly toxic and explosive reductant,^57–60,71^ and the need for soft templating for sufficient alignment.^72^ The mortar gelation, on the other hand, achieves multiple times smaller ligaments of 30–40 nm without any special reaction conditions. Another difference in the literature is the often monolithic gel structure, whose strands have some degree of long-range order. The mortar gels, in contrast, are powderous with no long-range order. Their higher metal salt concentrations also facilitate more interconnectivity and the formation of nanochain bundles resembling double cones in addition to the parallel bundles (Figure 4B). All these considerations were only made for magnets placed before the reduction. However, the placement time, which has not yet been investigated, is equally important. If placed 3 s after adding water, the morphology is the same as without the magnet. At this point, the reduction is already completed, and randomly shaped microscopic gel fragments have formed. As a result, the magnet can only accelerate their further gelation but not introduce the same anisotropic alignment as before. For very short delays of 1 s, the morphology is a hybrid of the gels with and without a magnet. The alignment to parallel and double-cone bundles is still present, but significantly less pronounced. In addition, the strands are less straight and considerably shortened in length. Therefore, the moment of adding water is decisive for the morphology. The magnet also introduces the magnetic field strength as a new parameter. Increasing it slightly enlarges the ligaments, regardless of the placement time. The morphology and gelation time, on the other hand, remain unaffected (Figure S14). While an increase in NaBH_4_ Eq and H_2_O volume for the preceding magnet placement follows the same trend as without a magnet, the ligament size is mainly unaffected and stays around 35 nm for the 3-s-delayed placement. This suggests that the magnetic field overpowers the other two subtle trends, preventing both the formation of smaller ligaments due to enhanced NP fusion and gel precipitation, as well as larger ligaments due to anisotropic alignment (Figures S15, S16).

Finally, the three morphologies from Figure 4B and the conc. mortar method from Figure S13 were compared regarding their physicochemical properties (Figure 5). In accordance with the ligament size tendencies, the SSA and total pore volume (TPV) increase in the following order: hybrid gel, meandering network, nanochain bundles, and conc. mortar approach (Figure 5A). Considering the relatively large ligaments, this still leads to high SSAs of 20–30 m^2^/g. Although even higher values were reported, they contain substantial amounts of a secondary phase (presumably oxide) covering a major portion^12,71,72^ or the entire structure,^63,130^ contributing to the SSA and overestimating it. Typically, for metal aerogels, the isotherms (Figure 5B) show a mix of Type II/IV behavior, which is due to the coexistence of micro-, meso-, and macropores as interstices between the network strands.^131^ Notably, all isotherms contradict the BET theory. Although multilayer adsorption becomes less favorable as the relative pressure decreases, the hysteresis does not close at a value of 0.42. Commonly, a breathing effect of the entire aerogel network is assumed, where it swells during the adsorption and shrinks during the desorption of N_2_. This is supported by measurements and calculations by Reichenauer et al.^132,133^ In addition, the hysteresis is narrowed under a magnetic field. This corresponds to a shift from small to larger mesopores, as the bundle alignment creates larger interparticle spaces (Figure 5C). Also possible is the increased fusion of strands close to micro- and small mesopores due to compression by the magnetic field. These small pores are more likely bottleneck pores, which cause delayed N_2_ desorption and hysteresis. The XRD pattern (Figure 5E) of all aerogels confirms Fe as the only crystalline phase. Analog to Figure S1, a broad and intense background also indicates an amorphous part, presumably Fe, oxide, and oxide hydroxide. Notably, the nanochain bundle structure has the least intensive background. This coincides with the faster gelation time and enhanced crystallinity through anisotropic growth. The conc. mortar approach, in contrast, shows the largest proportion of amorphous phase, probably arising from the near-instant gelation and extremely high nucleation rate, which is less selective for crystallite formation. In XPS analysis (Figure 5D), however, Fe^0^ accounts for only a small proportion, while Fe^2+^ and Fe^3+^ dominate. This behavior is due to the lower information depth of XPS (10 nm) compared to XRD (few μm). With ligament sizes of 30–40 nm, this implies that the majority of the gel is formed of a metallic Fe core, covered in a smaller portion of an oxide passivation layer. This is consistent with the observations from TEM and SEM. The XPS spectra were deconvoluted for the Fe 2p orbitals to identify the chemical states. The peaks at 708.6 and 720.9 eV correlate to the 2p_3/2_ and 2p_1/2_ orbitals of Fe^0^, respectively. The peaks at 712.2 and 725.6 eV can be assigned to the 2p_3/2_ and 2p_1/2_ orbitals of Fe^2+^, and the peaks at 714.0 and 728.3 eV to Fe^3+^. The XPS spectra for the meandering, hybrid, and nanochain bundle morphologies are shown in Figure S17. All spectra show a similar distribution to that of the conc. mortar approach, except for the nanochain bundles, having a slightly larger proportion of Fe^0^.

**Figure 5:**
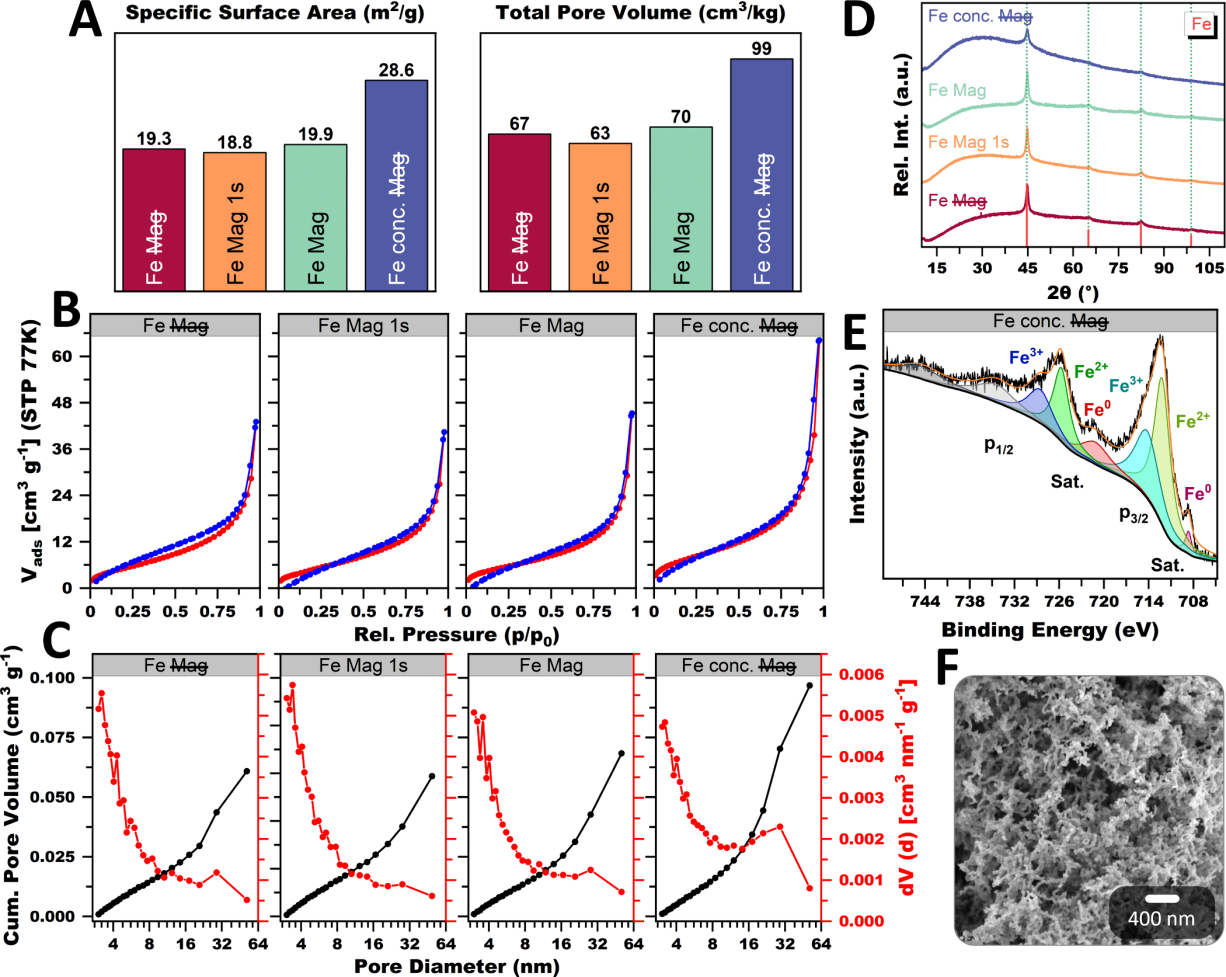
Physicochemical characterization of the three different Fe aerogel morphologies under standard mortar conditions and the conc. mortar approach. The N_2_ physisorption shows moderate SSAs and TPVs (A) for all Fe gels, following the ligament size trends observed before. These effects extend to the isotherm shape and pore size distribution (B, C). The hysteresis narrows in the following order: hybrid structure, meandering network, and nanochain bundles, due to a shift from small to larger mesopores. XRD (D) and XPS (F) confirmed the sample’s composition. The former shows a single crystalline Fe phase that forms the core of the aerogel, while the latter detects bi- and trivalent oxide species on the surface. An SEM micrograph (F) confirms the three-dimensional sponge structure of the gel.

### Other Metal Aerogels

To prove the versatility of the mortar approach beyond Fe, it was extended to all nonradioactive metals of groups 8–11, In, Sb, Pb, and Bi (Figure S18, Figure 6). The other tested elements are not reduced (Ga, Ge), form metal oxide gels (Zn, Cd), or bulk metals (Sn, Hg). A major difference from Fe is the longer gelation time, which is a few hours for base metals and metalloids, up to a day for noble metals. This correlates with their considerably less hygroscopic metal salts, leading to less complete partial reductions. Ni and Co additionally have lower reduction potentials. Special cases are Os and Ir: adding H_2_O initially turns the solution colorless, and after a few hours, black or blue. While Os gels overnight, Ir darkens for several days before forming a black gel with a blue tinge after 1 week. As before, standard mortar conditions were used and, if possible, the ungelled supernatant was discarded after 30 min. Even though it means a yield loss of approximately 20–40% for Rh, Ru, and Pt, or 5–10% for the other elements, good comparability to Fe is ensured. Moreover, the synthesis conditions could be adjusted analog to Figure 4A to enable complete gelation. The resulting morphologies differ drastically from Fe; the strand length is severely shortened, while the number of branching points increases. In places, it resembles a gel made of dendritic building blocks, especially for Co, Ni, Cu, Os, and Ir. The reason is a strong decrease in the dipole moment from Fe (1707 emu/cm^3^) to Co (1400 emu/cm^3^) to Ni (485 emu/cm^3^), while the other metals are not magnetic.^134^ As a result, the oriented attachment along a particular crystal plane is much less pronounced. Other element-specific features are aggregated spots for Rh, multilayered, sheet-like structures with internal aerogel morphology for Pd and Sb (Figure S19), and a needle-like secondary phase for In. Most metals exhibit small ligament sizes, with Ru, Os, Rh, Pd, and Ir exhibiting the smallest values between 2 and 5 nm. Pt, Ag, Bi, and Sb show moderate values between 8 and 21 nm. The largest ligaments are found for Au, Pb, and In, reaching up to 140 nm for Pb, whose metal salts are most hygroscopic. Although a broad inhomogeneity of differently shaped and sized ligaments remains, their distribution is narrower than that for Fe.

**Figure 6:**
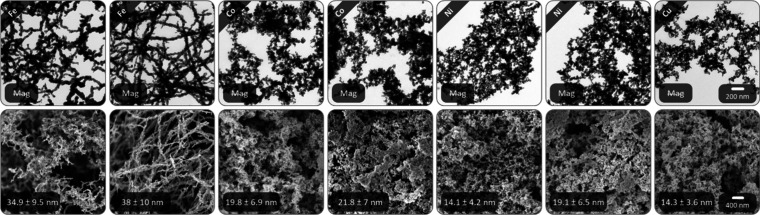
TEM and SEM micrographs of Fe, Co, Ni, and Cu base metal aerogels synthesized using the up-scaled mortar approach, each without and with an external magnetic field. The network structure remains the same compared to the standard conditions. However, the aerogels prepared in the presence of a magnetic field have more aggregated sites.

The SSA of the various elements roughly increases with decreasing ligament size, ranging from 1.8 to 83.2 m^2^/g (Figure S20). Pb and Au have particularly low values due to their highly hygroscopic metal salts, which are comparable to Fe. They attract more moisture in the partial reduction, intensifying aggregation and thus the formation of larger ligaments and more compact gels. The highest SSAs, on the other hand, are shown by gels with the longest gelation times: Pd, Rh, and Os. The TPV follows the same considerations. The ligament size and morphology in turn greatly influence the isotherm shape and pore size distribution (Figure S21). The hysteresis is wider for Au, Pb, and Rh. While the compact structures of Au and Pb suppress larger mesopores, interparticle spaces still allow for small mesopores. For Rh the aggregated spots disrupt the gel structure and micropores form around them. Ag, In, and Bi lie in the middle of these two extremes. Pt continues this trend, while its hysteresis is almost absent. For Pd and Sb, the rather dense multilayered sheet-like structure largely suppresses the formation of micro- and small mesopores and thus hysteresis. The dendritic network of Os causes a similar behavior by sterically favoring larger mesopores. XRD analysis (Figure S22) confirms the formation of crystalline phases of the respective element. While Rh, Pd, Pt, Ag, Au, and Pb have a cubic crystal structure, Sb and Bi are rhombohedral, In is tetragonal, and Os and Ru are hexagonal. Pb further forms PbO_2_ and PbO_*x*_ secondary phases. The Bragg peaks of Ir are split into doublets and shifted toward smaller angles. This is likely attributed to the coexistence of metallic Ir and IrO_2_ which forms via hydrolysis,^135^ the incorporation of rhombohedral boron in the cubic Ir lattice,^136^ and an increased concentration of lattice expanding defects.^137^ Ru has a particularly broad Bragg peak, covering three references. While this is consistent with the Scherrer equation and reflects the aerogel’s very small ligament size,^138,139^ the presence of an amorphous phase has an even greater impact.^140,141^ In amorphous phases, the absence of long-range order results in diffuse scattering, as various short-range motifs overlap, leading to an analog but more pronounced broadening. Similar features, albeit less pronounced, are also observed for most other elements.

The batch size is another important factor. Compared to Liu’s original one-step gelation, the conc. mortar approach reduces the H_2_O volume from 400 to 2 mL, i.e., a 200-fold increase in metal salt concentration. However, the set amount of 0.1 mmol metal salt limits the yield. Therefore, the batch size was increased 100-fold for all base metals. The resulting TEM and SEM micrographs (Figure 6) show the same morphology as before, perhaps slightly less compact and aggregated due to the larger solvent volume and grounding distance. While the ligament sizes of Fe remain almost identical to the standard approach, those of Co and Ni slightly increase regardless of the placement time of the magnet. Cu shows an inverse trend and the ligament size decreases. In general, the ligament size also increases when a magnetic field is applied, and more aggregates form for Co and Ni. While Co still shows sporadic nanochain bundles, albeit significantly shorter and less straight than Fe, they are entirely absent for Ni. The remaining structure is identical to the sample without magnetic field. Although the magnet does not influence the morphology of Co and Ni, it shortens their gelation time to around 1 h.

By simply grinding multiple metal salts at once, bimetallic aerogels are possible. This was demonstrated with Fe–Co, Fe–Ni, and Fe–Cu. They show morphologies that represent the structural features of both metals (Figure 7). Depending on the presence of a magnetic field, Fe causes the formation of elongated gel strands or nanochain bundles. The other base metals increase the number of branching points, shorten the strand length, make it less straight, and the bundle character less pronounced. The ligament size decreases analog to the monometals in the order of Fe–Co, Fe–Ni, and Fe–Cu and increases slightly when a magnetic field is applied. STEM-EDX-based element mapping for Fe–Ni gels (Figure 7) further reveal a nonuniform element distribution. In part, the distribution is homogeneous, but more often Ni shows increased abundance in the edge of the strand and Fe in the core. This is particularly pronounced for the synthesis under a magnetic field. Likewise, this is consistent with the lower reduction potential of Ni (−236 mV) compared to Fe (−37 mV),^142^ meaning a delayed Ni reduction, the effect of which is possibly pronounced even stronger due to the extremely short gelation time.

**Figure 7:**
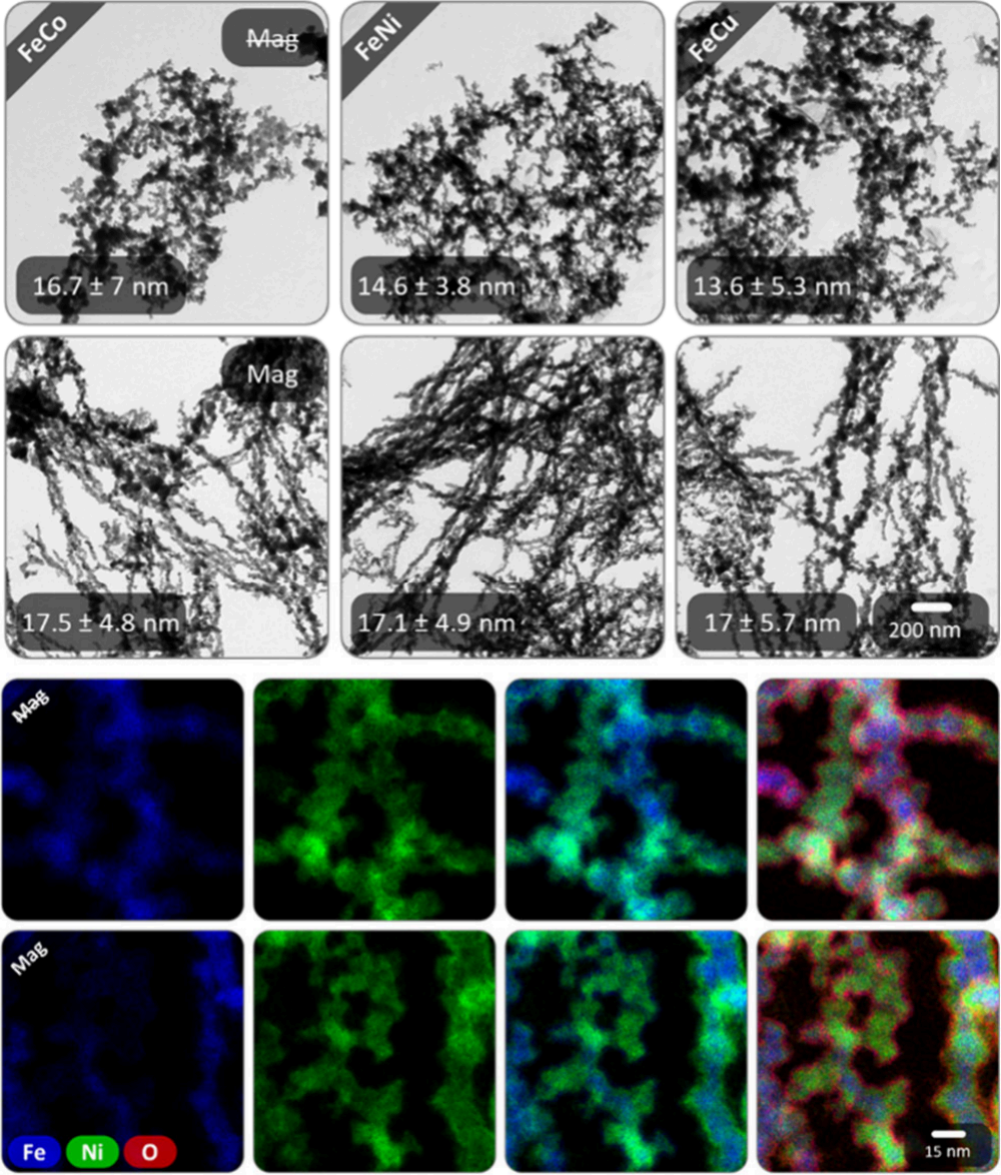
TEM micrographs of the bimetallic base-metal Fe–Co, Fe–Ni, and Fe–Cu aerogels in the absence and presence of an external magnetic field during the synthesis (top). The resulting structure combines the salient features of both respective metals. STEM-EDX-based element mapping of the Fe–Ni gels synthesized without and with magnetic field (bottom). The element distributions are rather nonuniform and with a higher abundance of Fe in the core and Ni in the edges of the strands, especially in the presence of a magnetic field.

Further bimetallic aerogels were prepared from Pd–Sb, Pd–In, Pd–Fe, Pd–Pt, Fe–Mo, and Fe–Cr (Figure S23). While some systems (Pd–Sb, Pd–Fe without magnet) homogeneously combine their individual morphologies analog to the bimetallic base metals, others (Pd–Pt) show segregated structures of gel compartments with vastly different ligament sizes. Fe–Mo and Fe–Cr show the same tendency as Pd–Pt but less pronounced. The magnetic field can introduce a similar separation in which Fe is seemingly aligned into nanochain bundles and Pd grows on top.

So far, only pure solvents have been used to complete the gelation. If a 30-ml Pd hydrogel suspension (0.05 mmol Pd) is used to synthesize an Fe aerogel instead, the Pd gel is mainly loaded onto the Fe network strands and, in a small amount, perhaps even integrated (Figure S24). This is independent of the presence of a magnetic field. The resulting heterostructure aerogel combines the positive effects of both gels, with the Fe component acting as a magnetic carrier. The structure is validated by STEM-EDX-based element mapping for a Pd@Fe gel synthesized without magnetic field (Figure S25). While Fe forms a pure overarching network, Pd forms small gel fragments with smaller ligament size which deposit on the Fe strands. Occasionally the Pd gel fragments can also get incorporated into an Fe strand. However, this is rarely observed for individual NPs.

A similar loading is possible by successive mortar synthesis (Figure S24). In a first mortar synthesis, a sol/small gel fragment solution is prepared and used as a solvent substitute in a second mortar synthesis. This is exemplified by Cu loaded on Fe. Compared to Pd, Cu is spread more evenly, as the in situ generated gel fragments are smaller than in the Pd suspension.

The physicochemical properties of the up-scaled base metal aerogels are summarized in Figure 8. The SSA values (Figure 8A) mainly decrease in accordance with the respective ligament size and in the order of Fe, Ni, Co, and Cu. An exception is Fe, which has the highest values, although it has by far the largest ligaments. This could be due to more compact and aggregated structures of Co, Ni, and Cu, leading to the overlapping of gel compartments and partial blockage of gas adsorption on the surface. Under a magnetic field, this behavior is even more pronounced, leading to further reduction of the SSA. As before, the TPV largely follows the SSA trends. The physisorption isotherms (Figure S26) also show the same mix of Type II/IV behavior and breathing effect as Fe. In addition, the magnetic field slightly broadens the hysteresis, correlating with a subtle shift to smaller mesopores. This is likely due to larger batch sizes, whereby the magnetic field and, thus, gel compression are weakened over greater distances. For Cu, larger mesopores dominate, analog to Os. Furthermore, XRD analysis (Figure 8B) confirms the formation of the respective crystalline base metal phases. Only Cu has a small additional proportion of Cu_2_O. Broad and intense backgrounds also indicate the formation of an amorphous phase. This is particularly prominent for Co. As before, XPS analysis (Figure 8C) shows a minor proportion of Fe^0^, while the oxidic species dominate. However, the metallic content of the surface significantly increases in the order Co, Ni, and Cu. For Cu, it is almost exclusively metallic. This aligns with the reactivity series of the elements, which follows the same order, meaning that oxidation gets ever less favorable.^143^ This is also observable in TEM as the passivation layer of the aerogel gets thinner. All XPS spectra show a high- and low-energy band corresponding to the 2p_1/2_ and 2p_3/2_ orbitals, which are trailed by satellite peaks on the higher binding energy side. When deconvoluted, for the Fe spectra, the peaks at 708.7 and 720.7 eV are assigned to Fe^0^, the peaks at 712.5 and 725.9 eV to Fe^2+^, and the peaks at 714.2 and 728.4 eV to Fe^3+^, for the 2p_3/2_ and 2p_1/2_ orbitals, respectively.^129^ The Co spectra can be deconvoluted in the peaks at 780 and 795.1 eV for Co^0^, 783.3 and 799.5 eV for Co^2+^, and 788.1 and 805.3 eV for satellite peaks of the 2p_3/2_ and 2p_1/2_ orbitals, respectively.^62,130,144^ The spin–orbit split of the Co^2+^ peak and the satellite shakeup match the literature.^144^ The Ni spectra show peaks at 854.4 and 871.7 eV for Ni^0^, 858.1 and 875.8 eV for Ni^2+^, and 863.3 and 881.7 eV for the satellites of the 2p_3/2_ and 2p_1/2_ orbitals, respectively.^145,146^ Cu shows peaks at 933.8 and 935.8 eV for Cu^0^ and 953.65 and 955.91 eV for Cu^2+^ of the 2p_3/2_ and 2p_1/2_ orbitals, respectively.^35,147^ The XPS spectra of the aerogels synthesized within a magnetic field show the same peak positions and surface compositions (Figure S27).

**Figure 8:**
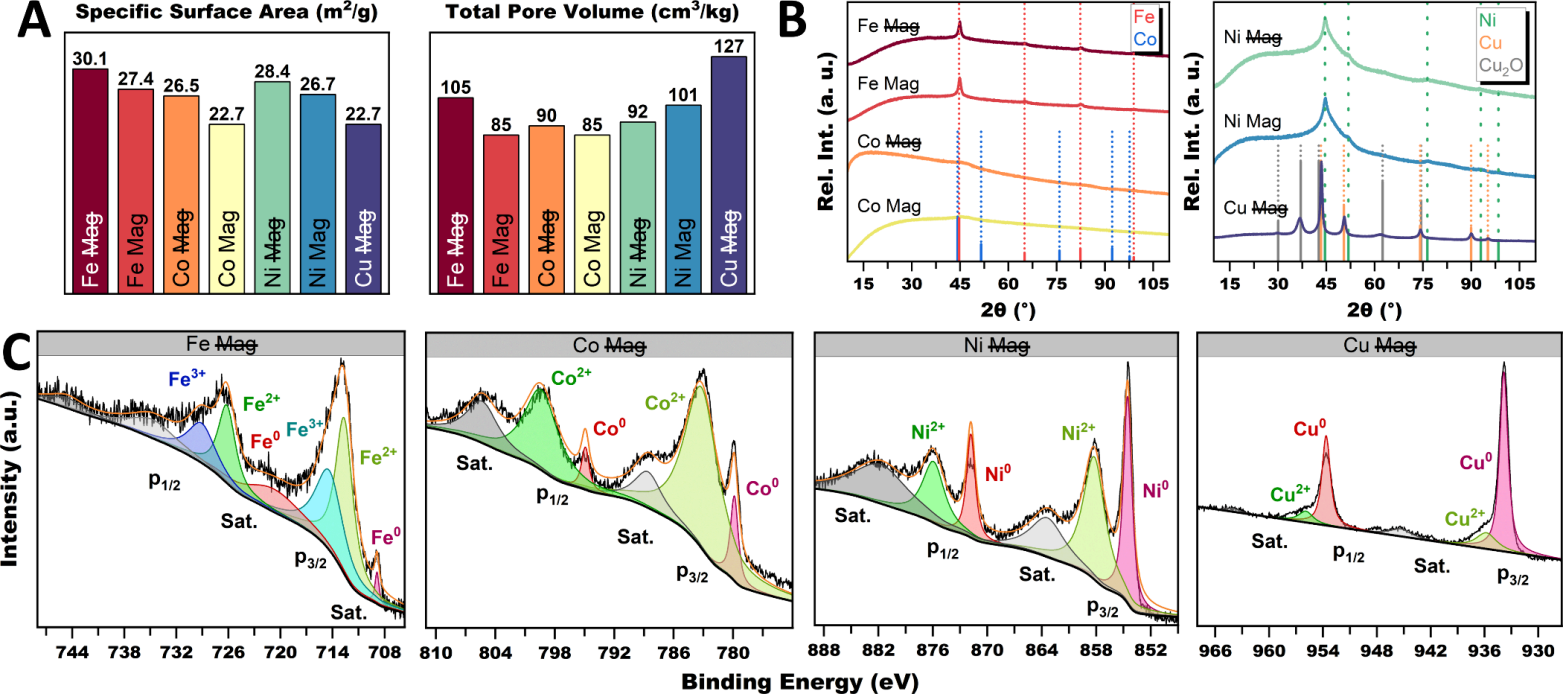
Physiochemical characterization of the Fe, Co, Ni, and Cu base metal aerogels under up-scaled mortar conditions. The N_2_ physisorption shows moderate SSAs and TPVs (A) for all base metal gels. When applying the magnetic field during the synthesis, the SSAs and TPVs slightly decrease. Due to the bigger batch size, mostly larger mesopores are reduced in size, broadening the hysteresis as more small pores are present in the aerogels. This slight shift from larger to smaller mesopores can be observed in the pore size distribution. The XRD patterns and XPS spectra conform to the composition of the gel, which are metallic gel strands covered with a passivation layer. Its thickness decreases with the reactivity series of the elements.

To test the limits of the mortar approach, the Pd precursor concentration was increased by up to 2000-fold compared to Liu’s method. The corresponding TEM micrographs (Figure 9D) show gels with the same sheet-like structure as before, but the higher the scaling factor, the more they increase in ligament size and compactness. This is reflected in a decrease in the SSA (Figure 9A), proportional to the scaling factor. Likewise, the number of micro- and small mesopores, representing the bottleneck pores that trap and delay N_2_ desorption, drastically decreases, making the hysteresis less pronounced (Figure 9B). Medium-sized mesopores, on the other hand, are only affected very slightly (Figure 9C). Remarkably, the scaling factor of 2000 uses as little as 1 mL H_2_O. We see no reason why the batch size could not be increased several orders of magnitude, enabling yields in the gram scale. This should also eliminate the issue of spontaneous inflammation when infusing a large quantity of ground powder with a tiny volume of H_2_O. It can be assumed that the reaction energy released is sufficient to ignite (also repeatedly) either the in situ generated H_2_ when mixed with air or NaBH_4_ at the dry edges of the mortar. When all solids are submerged in water, it absorbs the energy and suppresses ignition. This burning behavior considerably influences the nanostructure as seen in XRD (Figure 9E). At a scaling factor of 200, only one crystalline phase is present, and all Bragg reflections match the reference peaks of Pd. However, as the scaling factor increases, the Bragg peaks develop a shoulder toward smaller diffraction angles. In the case of the high-angle Bragg reflections of the 2000 scaling factor, the Bragg peaks even split into a doublet. This indicates the formation of a crystalline PdH_*x*_ phase, as Pd is known to form such phases easily. The high hydride and H_2_ concentration during the synthesis, as well as increased temperatures in the case of ignition, further promote their formation.^148–150^

**Figure 9:**
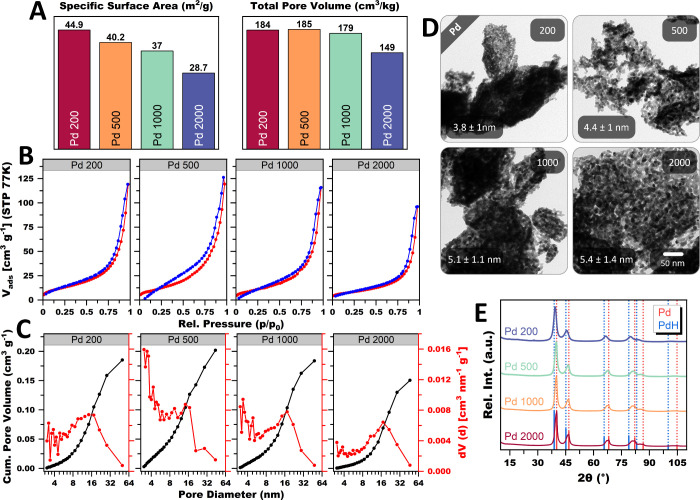
Physicochemical characterization of Pd aerogels synthesized by the mortar approach with metal concentration scaling factors of 200 (0.1 mmol PdCl_2_ infused by 2 mL H_2_O), 500 (0.25 mmol PdCl_2_ infused by 2 mL H_2_O), 1000 (0.5 mmol PdCl_2_ infused by 2 mL H_2_O), and 2000 (0.5 mmol PdCl_2_ infused by 1 mL H_2_O). With increasing the metal concentration, the SSAs and TPVs decrease steadily (A). This correlates with increased ligament size and compactness of the gel network, as shown in the TEM micrographs (B). The higher concentration also causes a narrowing of the hysteresis and a shift of the small mesopores to larger ones (C, D). All aerogels consist of a crystalline Pd phase and a minor fraction of PdH or intercalated hydrogen; the latter two increase with the Pd concentration.

## Conclusion and Outlook

The mortar synthesis is a highly flexible, versatile, scalable, and often instantaneous gelation method for a multitude of noble metals, base metals, and metalloids. To the best of our knowledge, this includes monometallic In, Sb, Pb, and Bi for the first time. Moreover, mortar synthesis achieves the fastest gelation and highest yield reported to date while also operating at the highest metal precursor concentrations so far, mostly with minimal trade-offs in ligament size and SSA. This is possible due to stepwise reduction by mechanochemical activation, followed by adding water, which decreases the effective metal concentration reduced in each step. The ligament size and SSA range from 1.9 nm for Os to 138 nm for Pb and 1.8 m^2^/g for Pb and 83.2 m^2^/g for In, respectively, with most gels exhibiting average values of 30–40 m^2^/g. Although the Pd metal salt concentration had already been increased 2000-fold compared to Liu’s original one-step gelation, we see no reason why the concentration and batch size could not be increased even further, or another metal could not substitute Pd. This enables gel production on a gram scale. While we have focused on mortar grinding to keep the synthesis as simple and accessible as possible, implementing ball milling could be very attractive for future up-scaling. It offers the potential for significantly increasing yield, reproducibility, precision, and experimental control due to the numerous ball mill-specific parameters and their existing industrial adaption. However, this comes with increased complexity, and many initial adaption challenges (risk of galvanic exchange, integrate flash-milling, integrate B-field, gas-generating reductants, control gaseous environment in glovebox, etc.), warranting a dedicated study on its own. While we have focused on monometallic systems, we also demonstrated the gelation of bimetals and investigated their element distribution. However, a detailed study of multimetallic aerogels and elements-specific properties such as prolonged stabilization (Os, Ir), shock-sensitivity (Ru), and sheet-like structures (Pd, Sb) is material for future studies. Heterostructures were also just briefly mentioned, but offer a unique way of loading metal gel fragments onto another metal gel. The presence of a magnetic field considerably shortens the gelation and washing times, but most importantly, it determines the morphology of the Fe gels: nanochain bundles, random networks, and hybrids. Co and Ni gels hardly form straight nanochains and may require stronger magnets to achieve similar structuring. The ligament size tends to subtly decrease with increasing residence time in H_2_O, H_2_O volume, grinding time, and NaBH_4_ Eq, while the Na_3_Cit amount, EtOH percentage in H_2_O, adhesive force of the magnet, and pH have the opposite effect. These parameters are interdependent and can lead to synergistic effects, as shown for the NaBH_4_ Eq and H_2_O volume, as well as the Na_3_Cit amount and EtOH percentage. Machine learning could be a powerful tool to help identify more such synergies and transfer them between different systems.

## Supplementary Material





## Notes

Video of the mortar-based synthesis of Pd aerogel (MP4)
Supplementary table to Figure 1 summarizing the reaction conditions of various metal aerogels reported in the literature. Photo series and corresponding XRD patterns of Fe aerogels synthesized under standard one-step, concentrated one-step, and mortar conditions; TEM micrographs of Fe aerogels under various reaction conditions with corresponding ligament size distributions and standard deviations; a table summarizing the influence of each synthesis parameter on the reaction behavior and morphology; XPS spectra of Fe aerogels with nanochain bundle, sponge-like, and hybrid morphologies; SEM and TEM micrographs, XRD patterns, and N_2_ physisorption of all other nonradioactive groups 8–11 metals, as well as In, Sb, Pb, Bi, and some of their bimetals (only TEM); N_2_ physisorption data and XPS spectra of concentrated Fe, Co, Ni, and Cu aerogels with and without magnetic field assistance; and TEM micrographs of Fe@Pd and Cu@Fe aerogels, including STEM-EDX elemental mapping for Pd@Fe (PDF)
