# Supplementary material for: Mortar Synthesis: A Mechanochemical Approach to Metal Aerogel Production
Source: Chem Mater. 2025 Oct 24;37(21):8707–23. doi: 10.1021/acs.chemmater.5c01607 (PMC12613324; doi:10.1021/acs.chemmater.5c01607)
Supplement: Supplementary file 2 [file cm5c01607_si_002.pdf]

Supporting Information

Mortar Synthesis: A Mechanochemical Approach to Metal Aerogel Production

Johannes Kresse[a,b], Laura Uhlmann[a,‡], Annika Christiansen[a,‡], René Hübner[c], Alexander Eychmüller[a*]

Author affiliation

[a] Physical Chemistry, TU Dresden, Zellescher Weg 19, 01069 Dresden, Germany

[b] Leibniz-Institut für Polymerforschung Dresden e. V., Hohe Straße 6, 01069 Dresden, Germany

[c] Institute of Ion Beam Physics and Materials Research, Helmholtz-Zentrum Dresden-Rossendorf e.V., 01328 Dresden, Germany

‡ both authors contributed equally

* E-Mail: Alexander.Eychmueller@tu-dresden.de

Table S1 Summary of the reaction conditions of various metal aerogels (blue), with particular focus on Pd (black) and Fe/base metals (yellow) as described in the literature. The corresponding abbreviations are defined below the table.

Strategy	Metal	Solvent	Reductant	Ligand	Additive	Temp. [°C]	Tag	Ref
Two-step gelation	PtAg	H_2O	$NaBH_4$	-	Centrifuge filter	RT	a	1
Substrate assisted two-step gelation	Au	H_2O	ascorbic acid	Na_3Cit, MBA	Au seeds (5 nm), APTES, polylysine	RT	b	2
One-step gelation	Pt_3Ni	H_2O	$NaBH_4$	-	-	RT	c	3
One-step gelation	Pt_3Ni	H_2O	$NaBH_4$	-	-	60	d	4
One-step gelation	AgPt	[APMIm]Br	N_2H_4	[APMIm]Br	Centrifugation	RT	e	5
One-step gelation	Ag	H_2O, EtOH, glycerol	N_2H_4	Glycerol	Thermostatic oscillator	0, 60	f	6
Oriented attachment	Pt	H_2O	KBH_4	Triton X-114	Centrifugation	0, 35	g	7
Disturbance	Au	H_2O	$NaBH_4$	-	Stirring	RT	h	8
Hydrothermal	Ru	H_2O	PVP	PVP, SDS	KF, centrifugation	180	i	9
Polyol	Ag	EG	EG, PVP	PVP	NaCl, $CuCl_2$	185	j	10
Polyol	Ag	EG	EG, PVP	PVP	$FeCl_3$	120, 150	k	11
Continuous	PtBi	H_2O	NH_3BH_3	PVP	HCl, centrifugation	60	l	12
One-step gelation	Pd	H_2O	$NaBH_4$	-	-	RT	m	13
One-step gelation	Pd	H_2O	$NaBH_4$	-	-	RT	n	14
One-step gelation	Pd	EtOH	$NaBH_4$	-	-	RT	o	15
Disturbance	Pd	H_2O	KBH_4	OP-10	Centrifugation	0	p	16
Soft templated one-step gelation	Pd	H_2O	DMAB	$C_{16}N$-Py	NH_4F, H_3BO_3, NH_3	50	q	17
Solvothermal	Pd	EtOH, glycerol	N_2H_4	Glycerol	-	180	r	18
Microwave	Pd	H_2O	$NaBH_4$	-	-	700 W (80 % Power)	s	19
Microwave	Pd	H_2O	Glyoxylic acid	Glyoxylic acid	Na_2CO_3	67.5	t	20
Soft templated radiolytic	Pd	H_2O, C_6H_{12}	In situ radicals	CTAB	Pentanol, centrifugation	RT	u	21
Polyol	Pd	DEG	DEG, PVP	PVP	-	140	v	22
One-step gelation	Fe	H_2O	$NaBH_4$	-	Solenoid	RT	w	23
One-step gelation	Fe	H_2O	$NaBH_4$	-	Nd magnet (under)	RT	x	24
One-step gelation	Fe	H_2O	$NaBH_4$	-	Ar gas, Nd magnets (flanking)	RT	y	25

One-step gelation	Fe	H_2O	$NaBH_4$	Dextran	Ar gas, Nd magnets (flanking)		z	26
One-step gelation	Fe	H_2O	$NaBH_4$	-	-	60	ä	27
Polyol	Fe	glycerol	N_2H_4	Glycerol, PVP	Centrifugation	reflux	ö	28
One-step gelation	Ni_8Fe_2	H_2O	N_2H_4	Na_3Cit	KOH, H_2PtCl_6, Helmholtz coil	RT, 80	ü	29
One-step gelation	FeCoNi	H_2O	$NaBH_4$	-	-	RT	á	30
One-step gelation	Ni	H_2O	N_2H_4	Na_3Cit	H_2PtCl_6, KOH, magnet (top, spinning)	RT	é	31
One-step gelation	Co	H_2O	N_2H_4	Na_2EDTA, PVP	NaOH, H_2PtCl_6, Nd magnets (flanking)	80	í	32
Two-step gelation	CuNi	H_2O	$NaBH_4$	Na_3Cit	NH_4Cl	RT	ó	33
Soft templated one-step gelation	Co	EG, H_2O	DMAB, $NaBH_4$	Pluronic F-127	Magnet (under)	RT, 40	ú	34
Freeze-casting	Cu	H_2O	N_2H_4	EDA, PVP	NaOH	80	ñ	35

Na_3Cit – Trisodium Citrate

APTES – (3-aminopropyl)triethoxysilane

Triton X-114 – p-(1,1,3,3-Tetramethylbutyl) phenyl-polyethylene glycol (7-8 C_2H_4O units)

SDS – sodium n-dodecyl sulfate

OP-10 – Polyethylene glycol p-(1,1,3,3-tetra-methylbutyl)- phenyl ether (≈10 C_2H_4O units)

C_{16}-py – Hexadecylpyridinium

DEG – Diethylene Glycol

Pluronic F-127 – α-Hydro-ω-hydroxypoly-(oxyethylene)-poly(oxypropylene)-poly-(oxyethylene) $(C_2H_4O)_{101}$–$(C_3H_6O)_{56}$–$(C_2H_4O)_{101}$

MBA – Mercaptobenzoic acid

[APMIm]Br – 1-Aminopropyl-3-methylimidazolium bromide

PVP – Polyvinylpyrrolidone

EG – Ethylene Glycol

DMAB – Dimethylamine Borane

CTAB – Cetyltrimethylammonium Bromide

Na_2EDTA – Disodium ethylenediaminetetraacetate

EDA – ethylenediamine

Video1.mp4

Video S1 Pd aerogel synthesis procedure under concentrated mortar conditions.

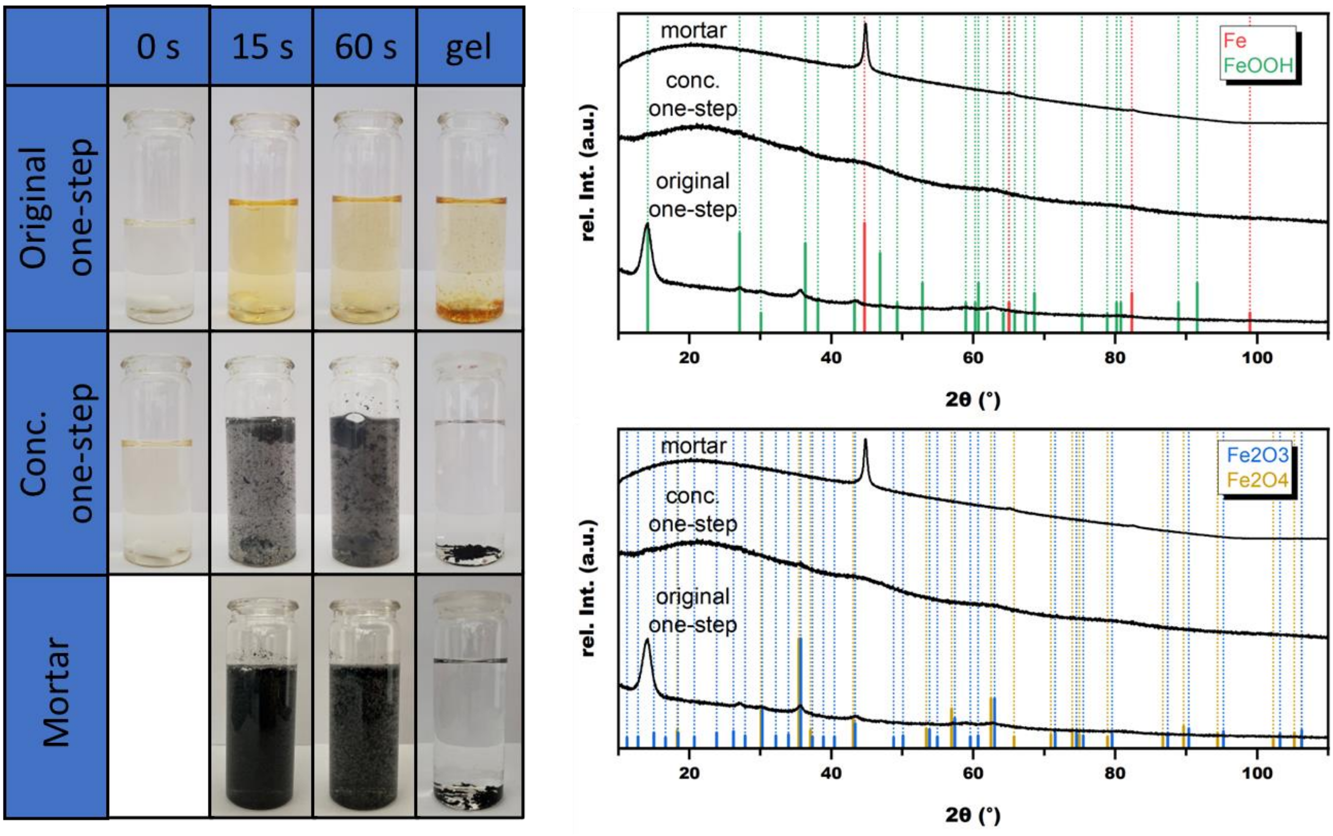

Figure S1 Photo series of the gelation process of Fe aerogels (left) prepared by the one-step gelation (6.25 µmol Fe^{3+} in 25 mL H_2O), concentrated one-step gelation (0.1 mmol Fe^{3+} in 30 mL H_2O), and mortar approach (0.1 mmol Fe^{3+} in 30 mL H_2O). Only the latter two methods result in a black gel, while the one-step approach forms an orange precipitate. The XRD patterns (right) show that only the mortar synthesis leads to a crystalline phase of metallic Fe. The orange precipitate is a mixture of iron oxide hydroxide and different oxides.

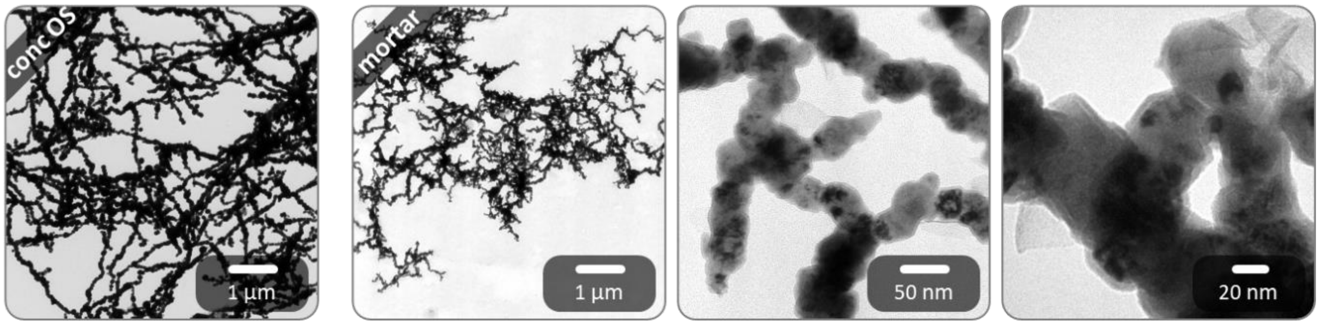

Figure S2 TEM micrographs of the concentrated one-step gelation (left) and the mortar synthesis (right) of Fe. Both gels show a comparable network structure, but the ligament size of the conc. one-step gelation (70 nm) is significantly higher than that of the mortar synthesis (30-40 nm). The latter network also shows an increased number of branching points.

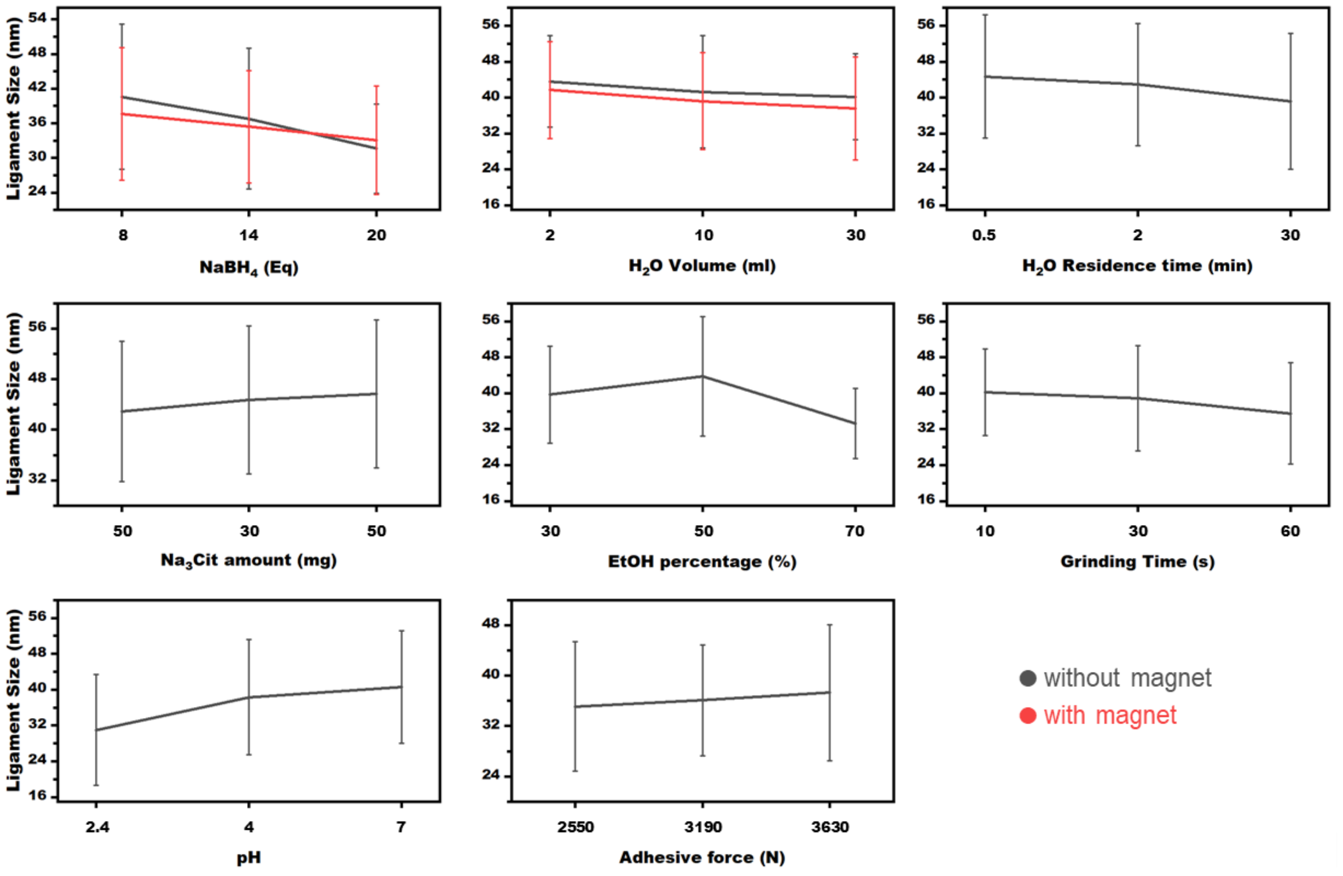

Figure S3 Summary of the various tendencies in ligament size, including standard deviation, as a function of different synthesis parameters. While an increase in the-H_2O residence time, H_2O volume, grinding time, and $NaBH_4$ amount tend to decrease the ligament size, an increase in the Na_3Cit amount, EtOH percentage, adhesive force of the magnet, and pH increase it.

Table S2 Summary of the various influences of the synthetic parameters on the gel properties alongside the ligament size. Increasing and decreasing parameters values are indicated by up/down arrows.

**Parameter ↑**	**Influence**	**Parameter ↓**	**Influence**
**Grinding sequence**	No morphological change	pH	Prevents oxidation, yield loss
**Grinding force**	No morphological change	$NaBH_4$ Eq	Faster grounding, less oxidation
**Grinding time**	Lumping of $NaBH_4$	H_2O volume	Faster grounding, less oxidation
**Residence time**	Promotes oxidation	Magnetic strength	No morphological change
**EtOH percentage**	Promotes oxidation	$NaBH_4$ Eq + Mag	Even faster grounding, less oxidation, nanobundle assembly
**Na_3Cit**	Prevents oxidation, smoother but less connected strands	H_2O Vol + Mag	Even faster grounding, less oxidation, nanobundle assembly

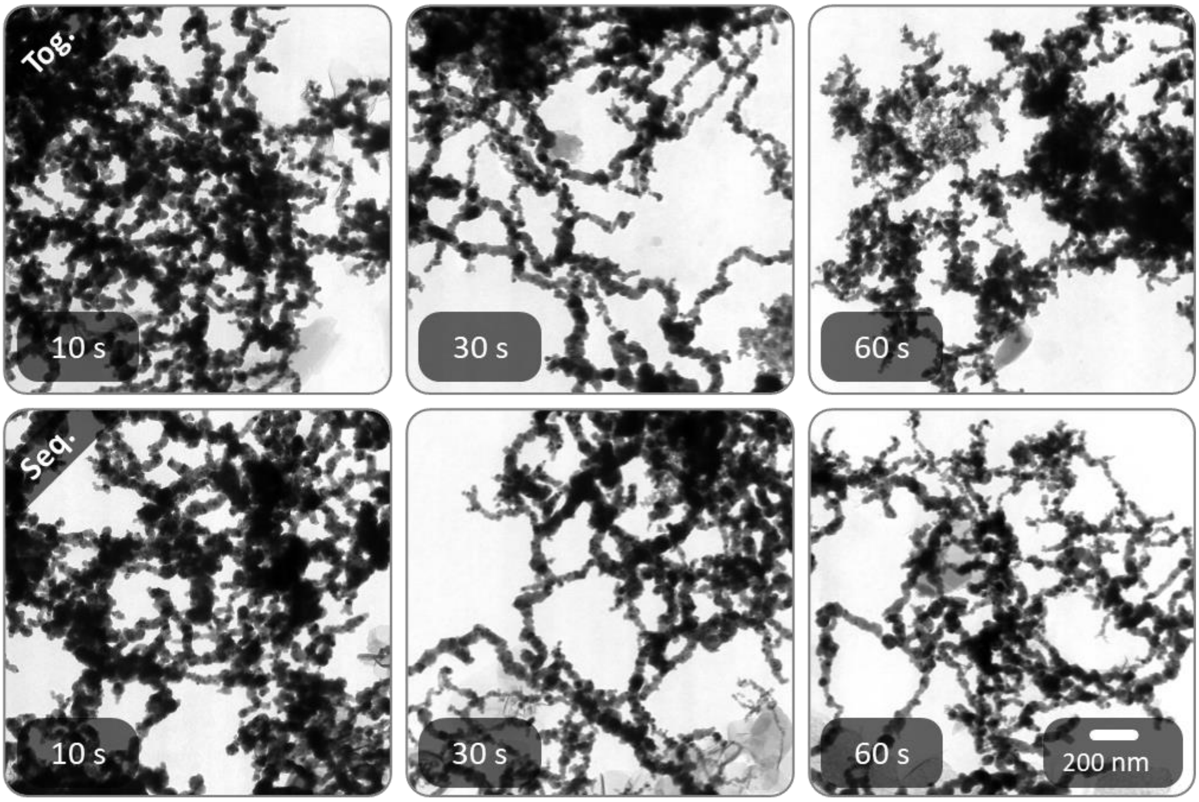

Figure S4 Fe aerogels synthesized with the standard mortar approach, where $FeCl_3$ and $NaBH_4$ are ground together at casual grinding force (top) and sequentially, first $NaBH_4$ then $NaBH_4$ and $FeCl_3$ combined at maximum grinding force (bottom), for 10 s (left), 30 s (middle), and 60 s (right). A prolonged grinding time slightly decreases the ligament size, while the grinding force and sequence have no significant influence on the ligament size and morphology.

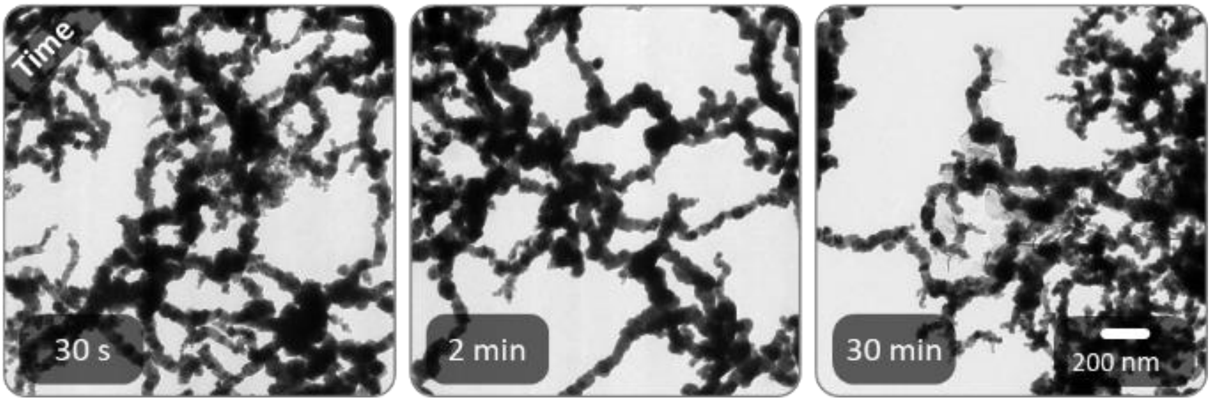

Figure S5 TEM micrographs of Fe aerogels synthesized under standard mortar conditions with residence times of 30 s, 2 min, and 30 min in H_2O. Although prolonged residence time does not influence the ligament size, it promotes the formation of secondary oxide structures.

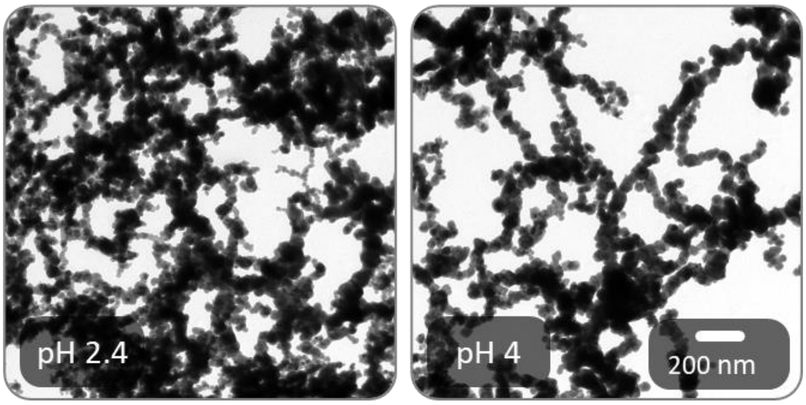

Figure S6 TEM micrographs of Fe aerogels under standard mortar conditions infused with water of a defined pH value. A lower pH value reduces the ligament size and prevents oxidation, but it also dissolves a significant proportion of the aerogel, reducing its yield. This is around 80 % for pH 2.4.

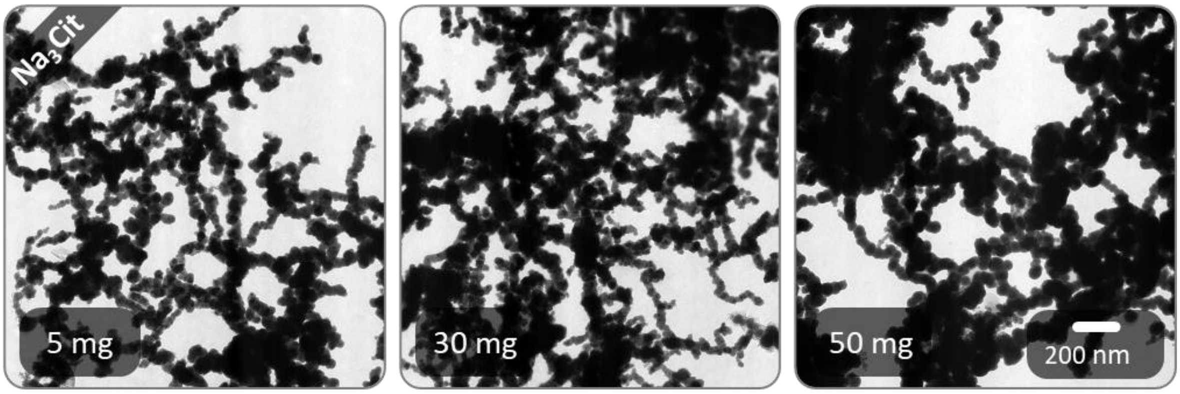

Figure S7 TEM micrographs of Fe aerogels synthesized using the standard mortar approach with the addition of 5 mg, 30 mg, and 50 mg of Na_3Cit when grinding $NaBH_4$. This suppresses oxidation but also increases the ligament size.

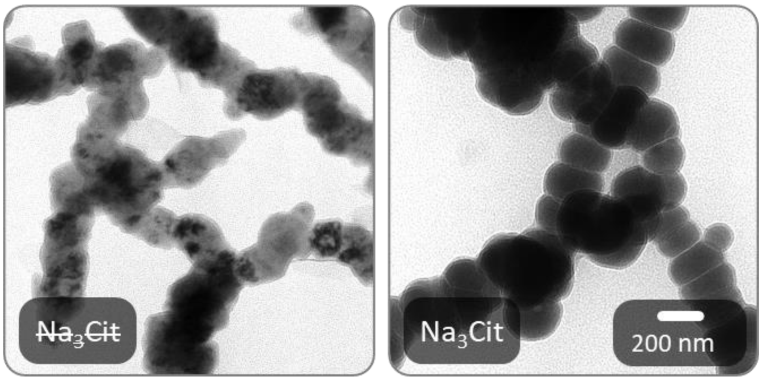

Figure S8 Comparison of high-magnification TEM micrographs for the standard mortar synthesis without and with 5 mg Na_3Cit additive. Both gels are covered with an equally sized passivation layer, but Na_3Cit suppresses secondary structure formation and causes fragmentation into smaller, more delimited compartments.

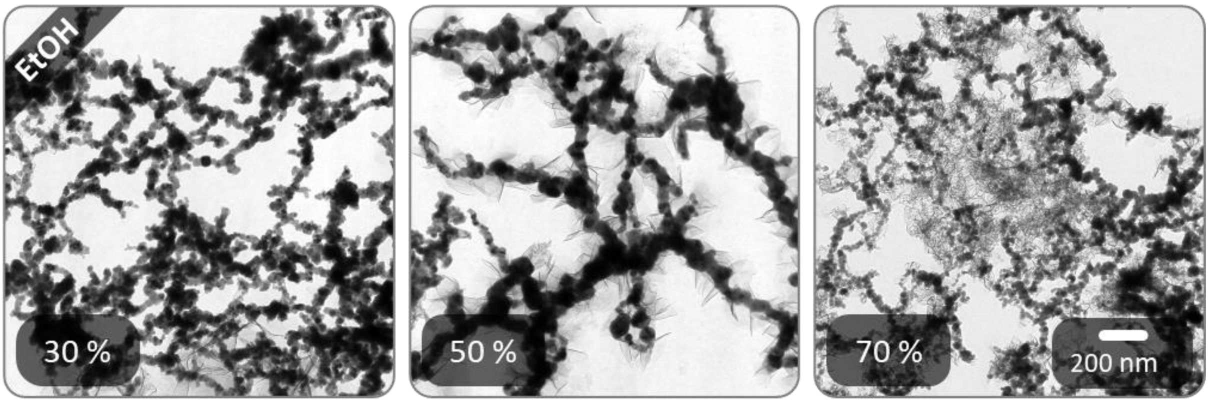

Figure S9 TEM micrographs of Fe aerogels from standard mortar conditions infused by water and ethanol solvent mixtures with an EtOH content of 30 %, 50 %, and 70 %. Initially, this prevents oxidation and decreases the ligament size, but this trend reverses as the EtOH content increases.

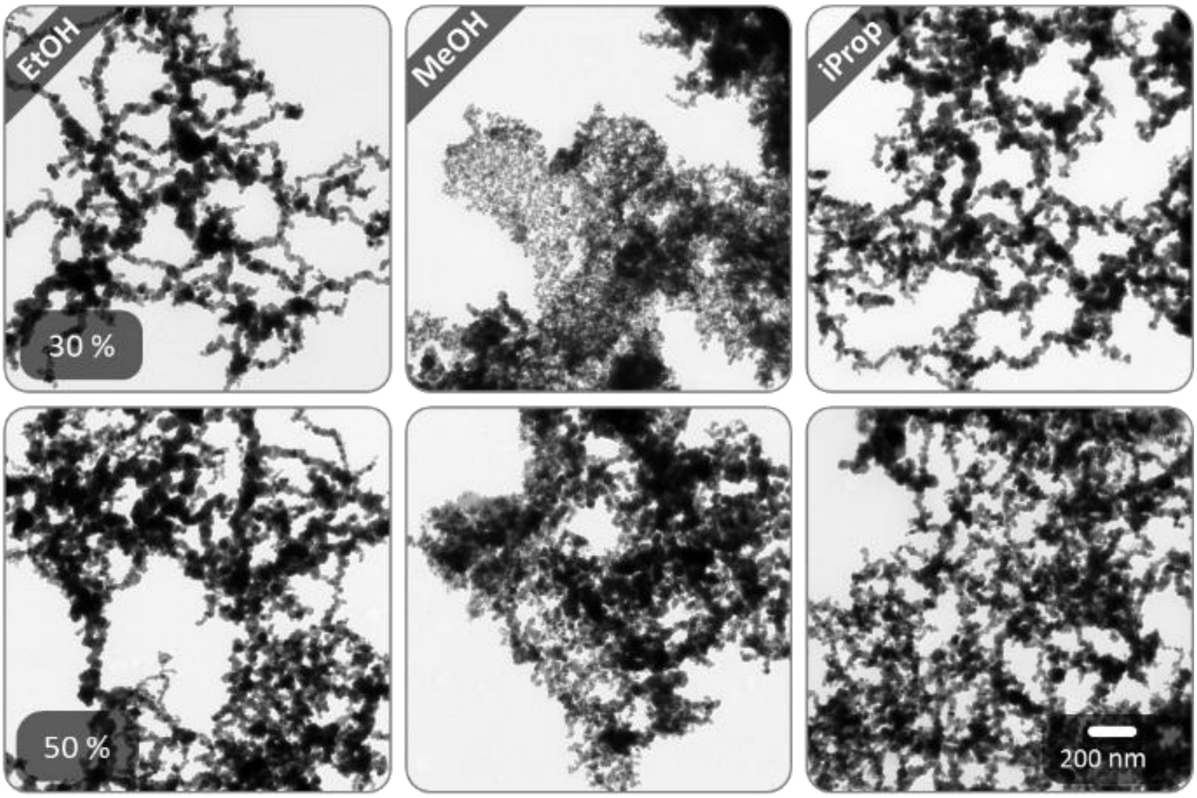

Figure S10 TEM micrographs of Fe aerogels under standard mortar conditions with 5 mg of Na_3Cit additive and alcohol (ethanol, methanol, isopropanol) water mixtures with an alcohol content of 30 % and 50 %. These two synthesis parameters have a synergistic effect that suppresses oxidation and reduces the ligament size. This does not apply to pure and high alcohol contents.

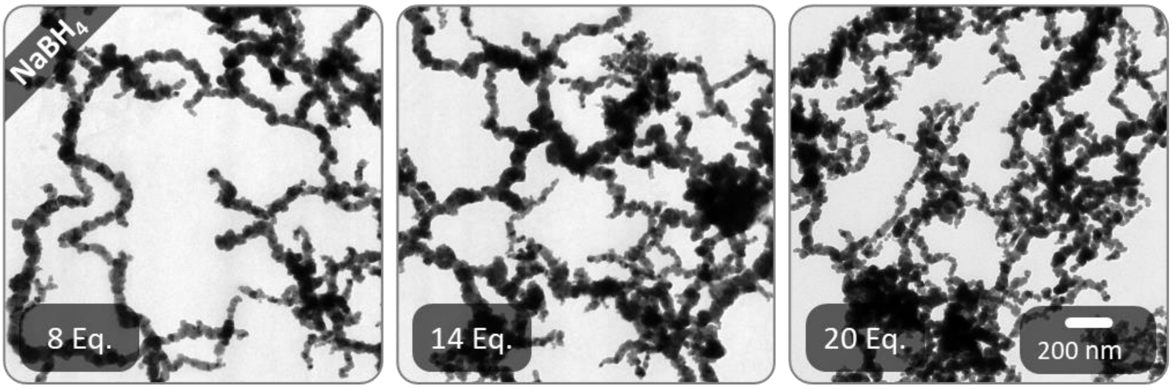

Figure S11 TEM micrographs of Fe aerogels synthesized under standard mortar conditions with $NaBH_4$ Eq of 8, 14, and 20. The gel network consists of randomly interconnected meandering nanochains of different sizes and shapes, which slightly decrease in ligament size with increasing $NaBH_4$ Eq.

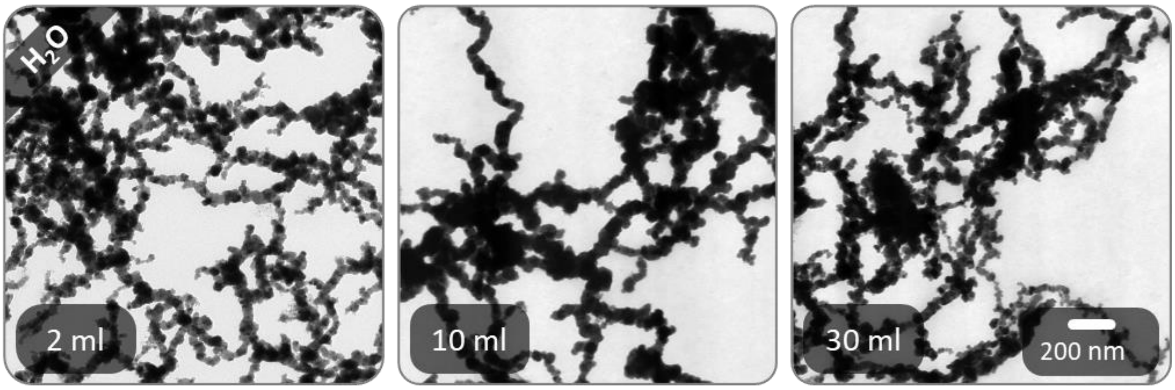

Figure S12 TEM micrographs of Fe aerogels synthesized under standard mortar conditions infused by 2, 10, and 30 mL water. The increasing volume slightly increases the ligament size.

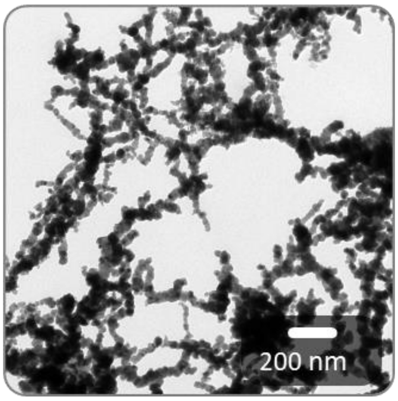

Figure S13 TEM micrograph of an Fe aerogel synthesized by using a conc. mortar approach (2 mL H_2O, 20 Eq. $NaBH_4$). The structure corresponds to the standard conditions, but the synergistic effect between the solvent volume and the $NaBH_4$ amount significantly decreases the ligament size.

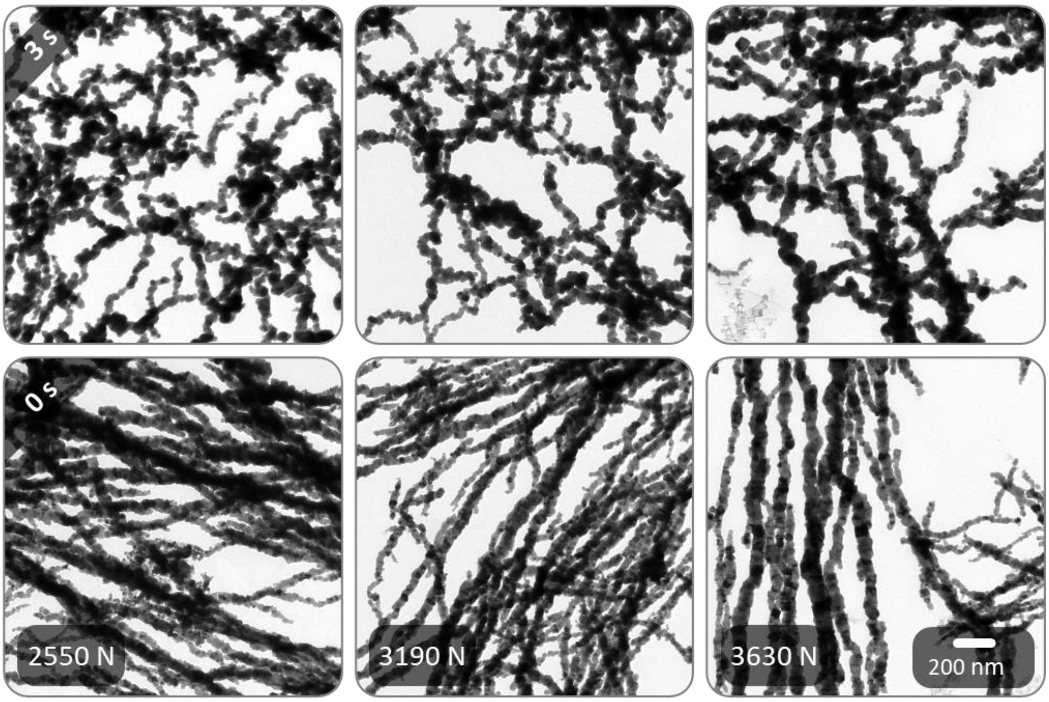

Figure S14 TEM micrographs of Fe aerogels synthesized under standard mortar conditions and application of external magnetic fields with adhesive forces of 2550 N, 3190 N, and 3630 N after 3 s (top) and before (bottom) adding water. The higher magnetic field strength increases the ligament size independent of the placement time of the magnet.

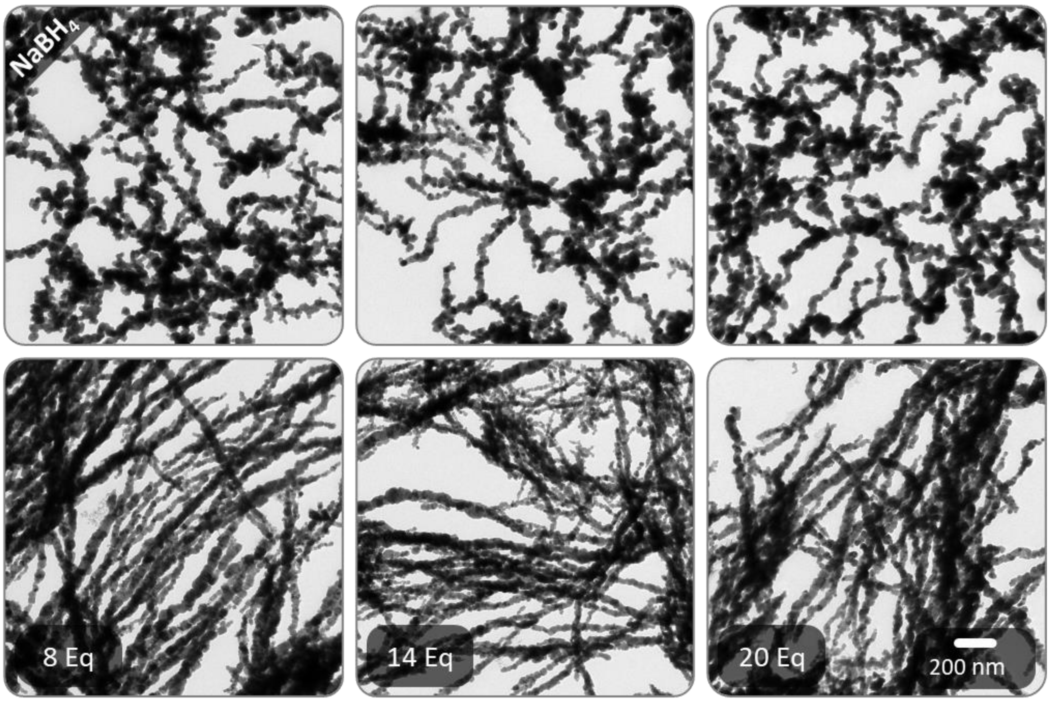

Figure S15 TEM micrographs of Fe aerogels synthesized using an external magnetic field of 2550 N adhesive force 3 s after (top) and before adding water (bottom). In the latter case, increasing the $NaBH_4$ Eq slightly decreases the ligament size similar to Figure S11. When the magnetic field is applied after 3 s, the size of the ligaments does not change significantly.

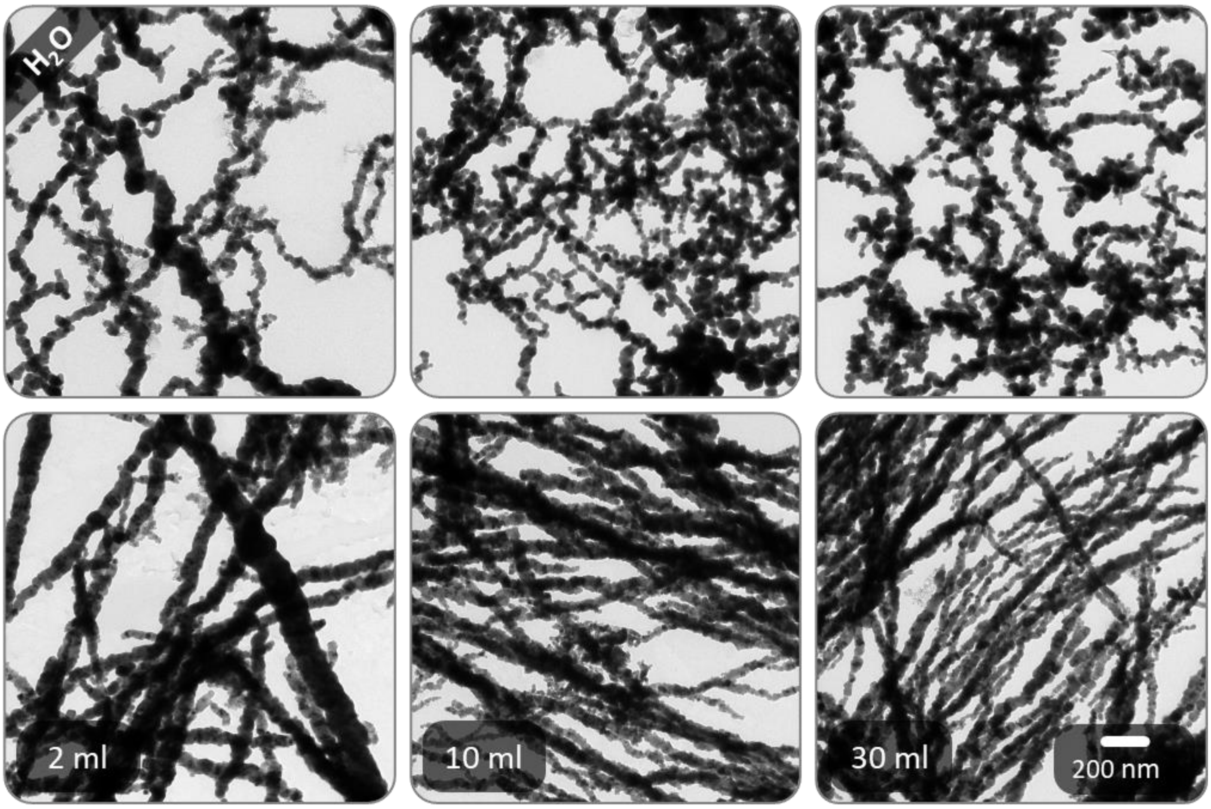

Figure S16 TEM micrographs of Fe aerogels synthesized using an external magnetic field of 2550 N adhesive force 3 s after (top) and before adding H_2O (bottom). In the latter case, increasing the H_2O volume slightly decreases the ligament size similar to Figure S12. When the magnetic field is applied after 3 s, the size of the ligaments does not change significantly.

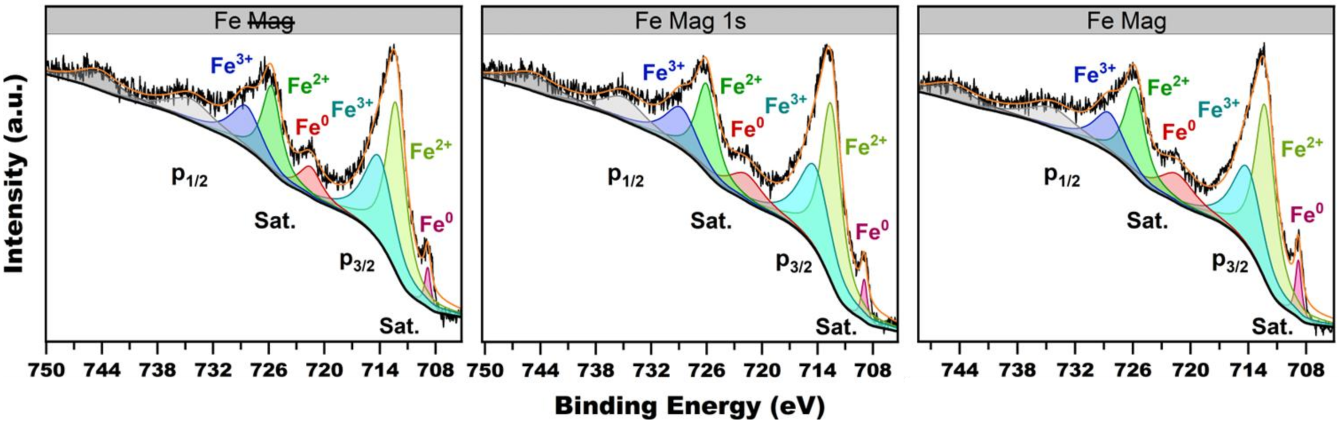

Figure S17 XPS spectra of Fe aerogels with three different morphologies: random meandering strands, hybrid structure, and nanochain bundles. The gels were synthesized under standard mortar conditions without the magnetic field, with a 1-s-delayed magnetic field, and with a magnetic field before adding water, respectively. All XPS spectra show a similar surface composition of the Fe aerogels, where bi- and trivalent oxidized species dominate and metallic Fe contributes only a small proportion. Notable, the nanochain bundles show a considerably higher content of metallic Fe than the other two morphologies.

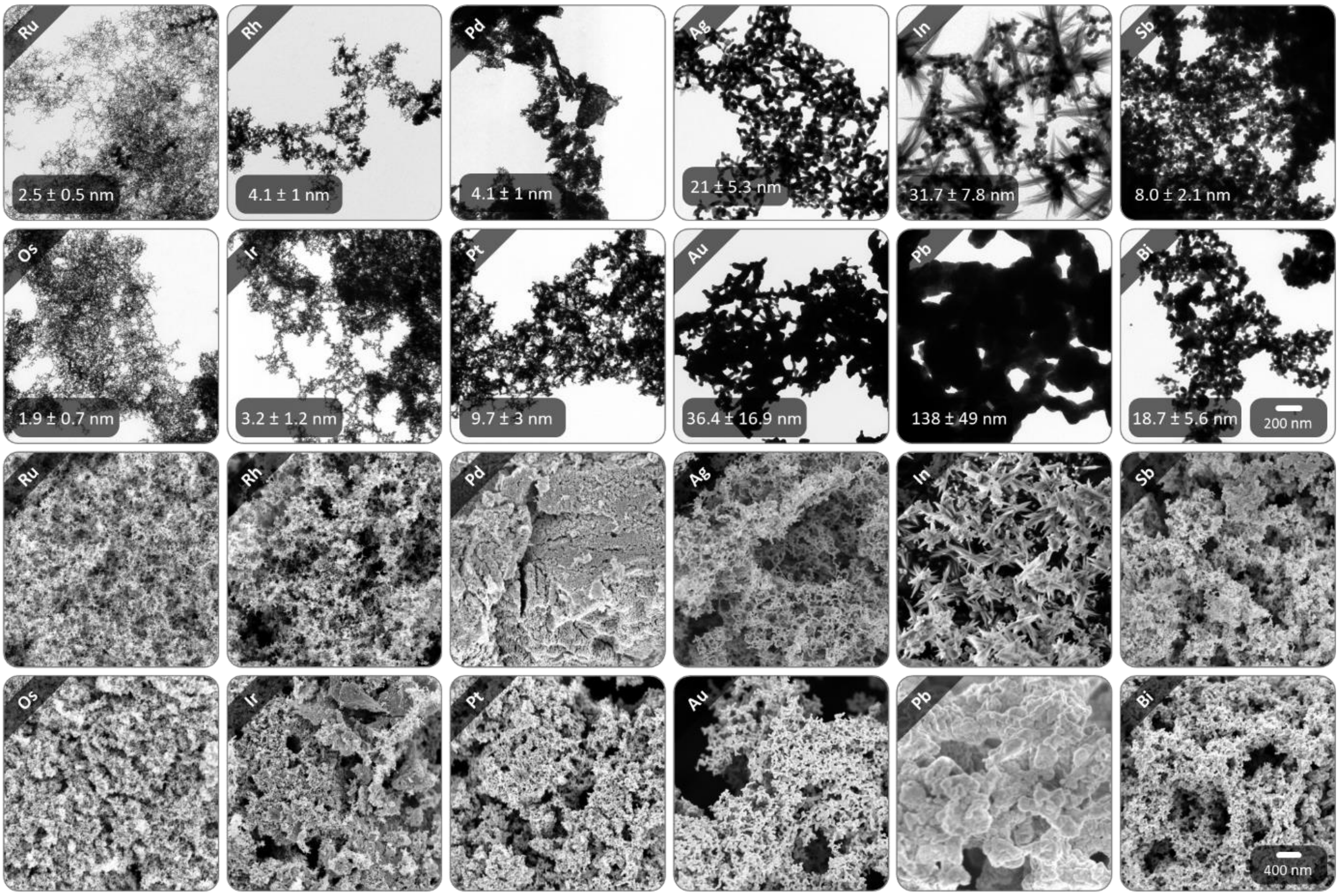

Figure S18 TEM and SEM micrographs of various noble metal and metalloid aerogels synthesized under standard mortar conditions. In addition to the typical aerogel structure, some systems also show element-specific morphologies, more precisely aggregated spots for Rh, a sheet-like structure for Pd and Sb, and a needle-shaped secondary structure for In. SEM micrographs further illustrate the three-dimensional porous nature of the aerogels.

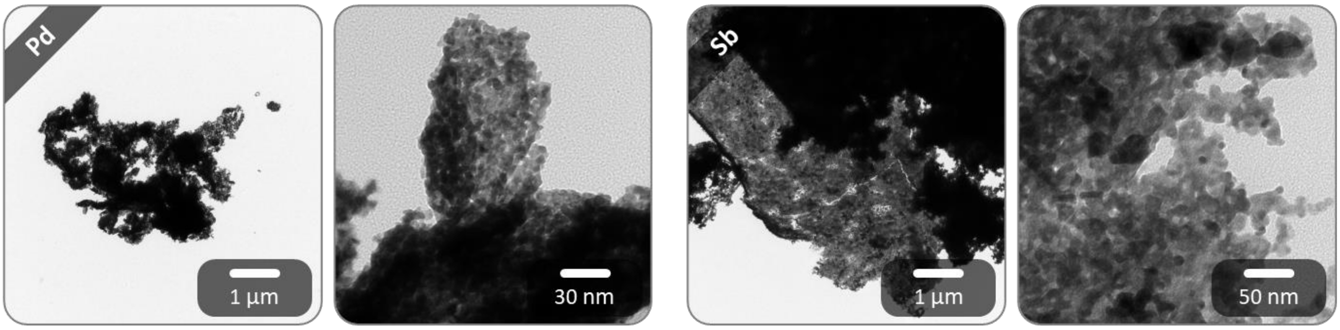

Figure S19 TEM micrographs of Pd and Sb aerogels highlighting the multilayered sheet-like structure.

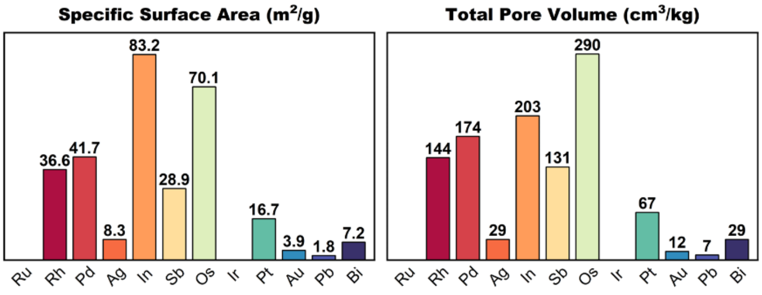

Figure S20 Comparison of the SSAs and TPVs of the noble metal and metalloid aerogels. Most of them show high to moderate values based on their small ligament sizes. Only Au and Pb show extremely low values due to the compact structures combined with larger ligaments. To some extent this also applies to Ag and Bi.

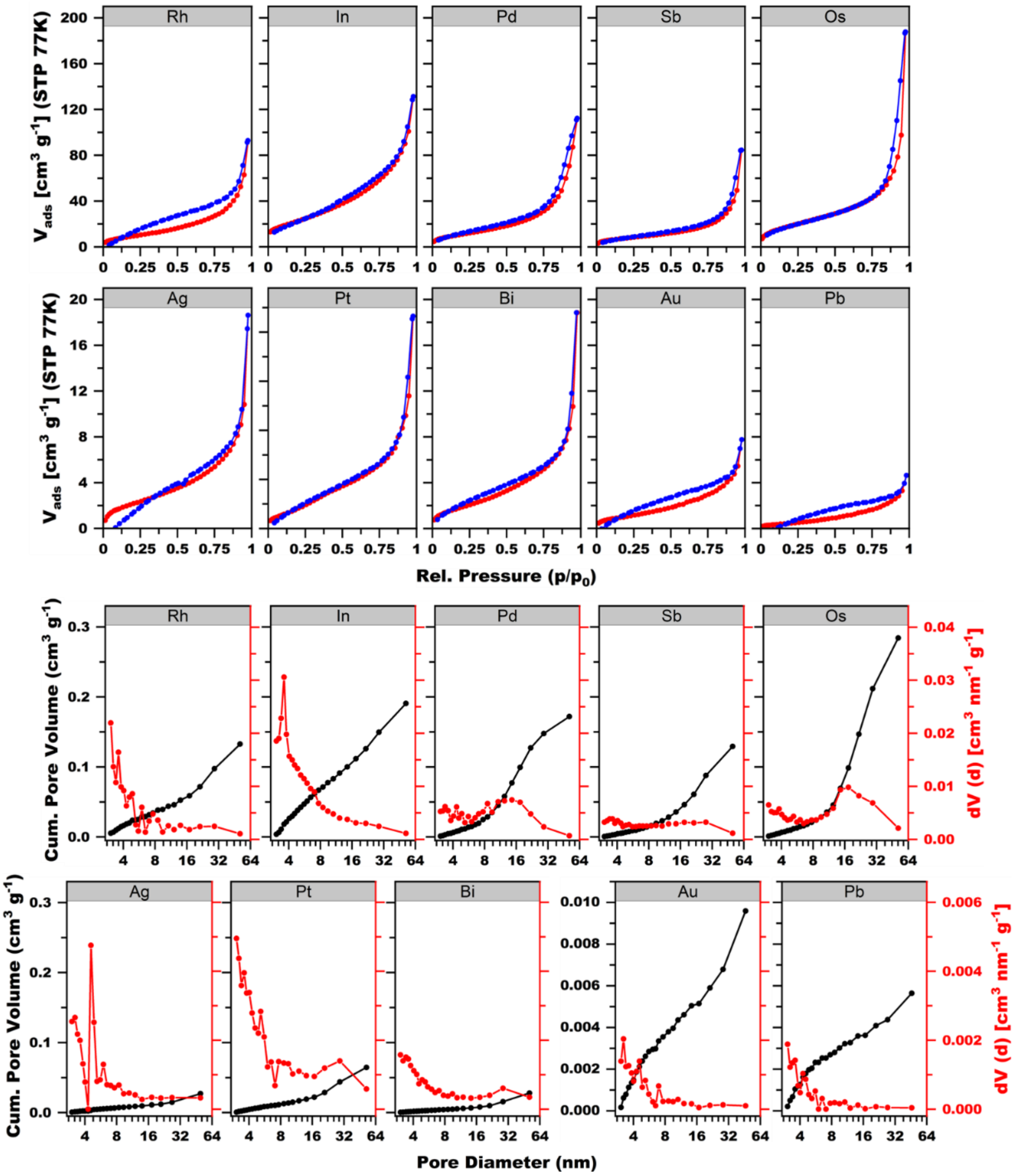

Figure S21 N_2 physisorption of the noble metal and metalloid aerogels. The isotherms and pore size distributions are fingerprints for each elements and morphology, linking the nanoscale structure observed by TEM imaging to the macroscopic porous properties.

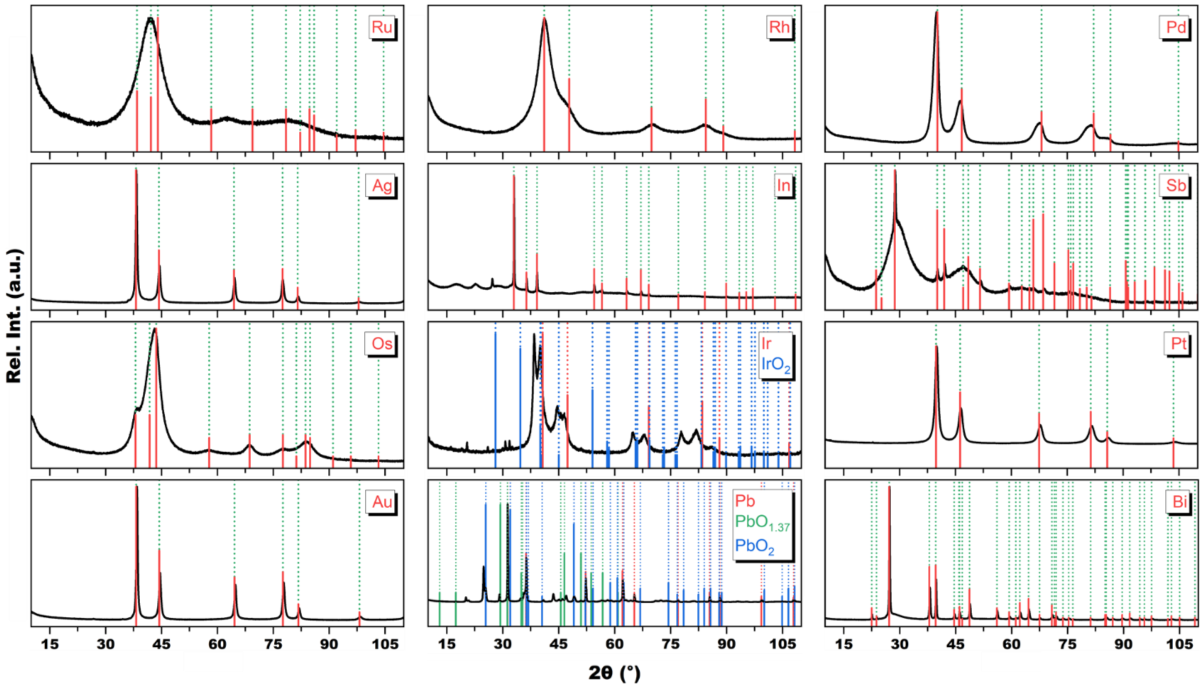

Figure S22 XRD patterns of the various noble metal and metalloid aerogels synthesized under standard mortar conditions. Except for Pb and Ir, which shows different oxidic side phases, all other aerogels show a pure phase of their respective element. In addition to a crystallin phase, there is also an amorphous phase, the proportion of which increases with the Bragg peak widths. Depending on the element, the crystal system can be cubic, hexagonal, or rhombohedral.

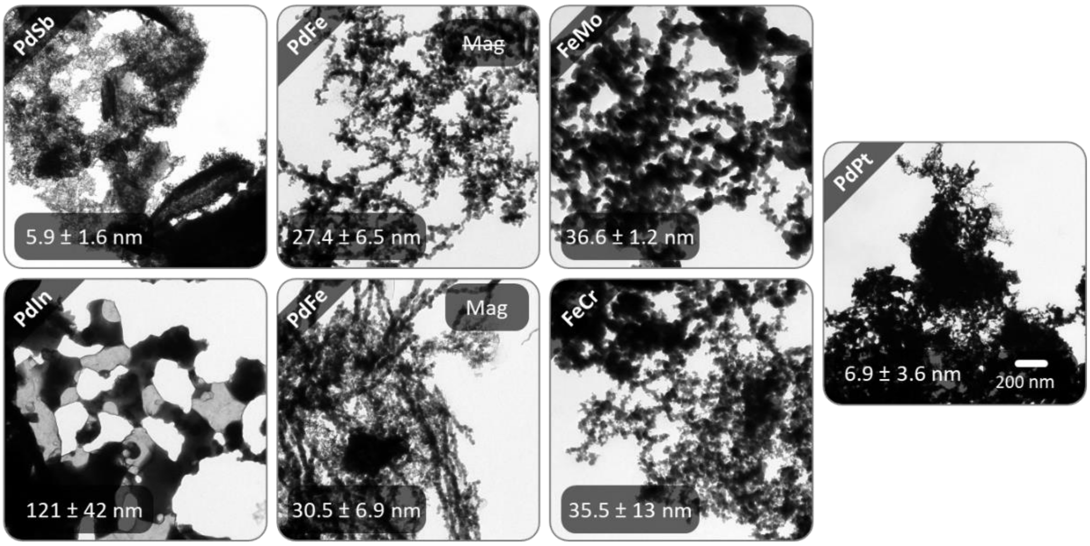

Figure S23 TEM micrographs of bimetallic Pd-Sb, Pd-In, and Pd-Fe (with and without external magnetic field), Fe-Mo Fe-Cr Pd-Pt aerogels synthesized under standard mortar conditions. Pd-Sb shows the typical aerogel structure, Pd-In alternating regions of extreme contrast difference, Pd-Fe has a similar structure to the bimetallic base metal aerogels, and Pd-Pt shows the typical aerogel structure but split into multiple regions of vastly different ligament size. The morphology of Fe-Mo and Fe-Cr aerogels is also comparable to that of the other bimetallic base metals, but their ligaments are more inhomogeneous, especially in the case of Fe-Cr, which consists of two networks with different sizes (9.1 ± 2.8 nm; 2.9 ± 1.0 nm).

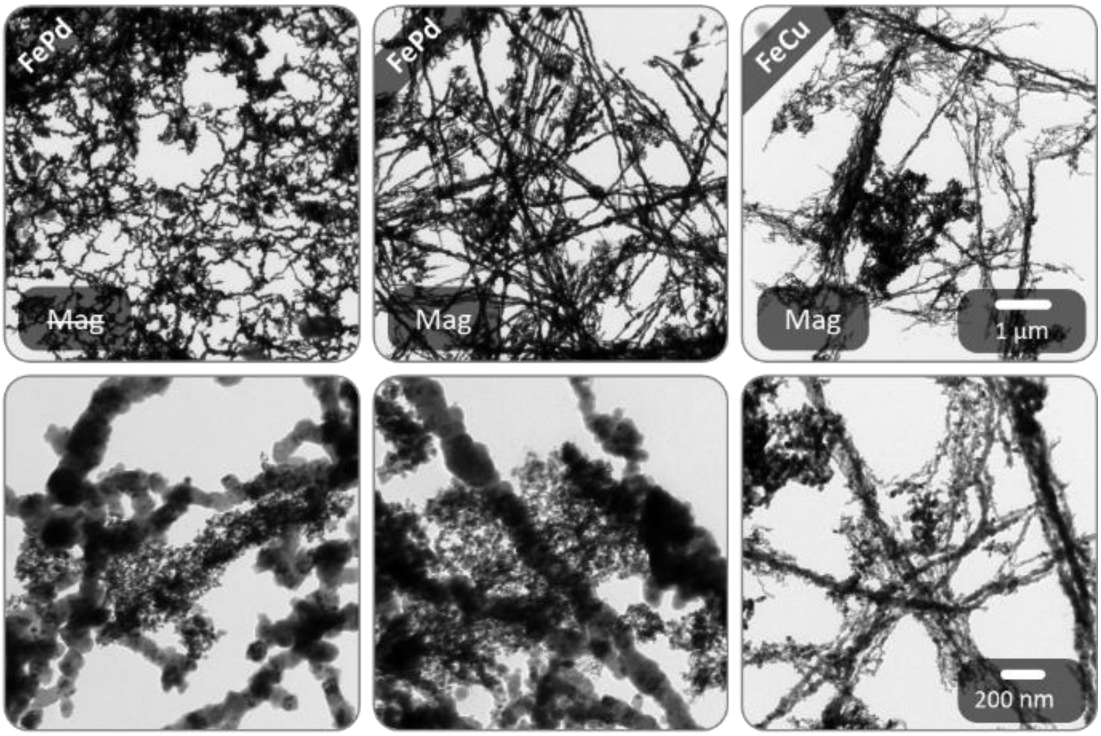

Figure S24 TEM micrographs of Pd and Cu gels loaded onto an in-situ forming Fe gel as part of the standard mortar synthesis. There are two loading pathways, either using a gel suspension (exemplified for Pd) or the non-gelled product of a mortar synthesis (exemplified for Cu) as a substitute for water as the reaction medium. The ligament sizes of the Fe-Pd structures without magnet are 36.4 ± 11.8 nm and 5 ± 1.2 nm, those of Fe-Pd with magnet are 39.2 ± 10.1 nm and 5.3 ± 1.2 nm, and those of Fe-Cu are 24.9 ± 10.4 nm and 17.4 ± 4.2 nm, respectively for the carrier gel and loaded gel.

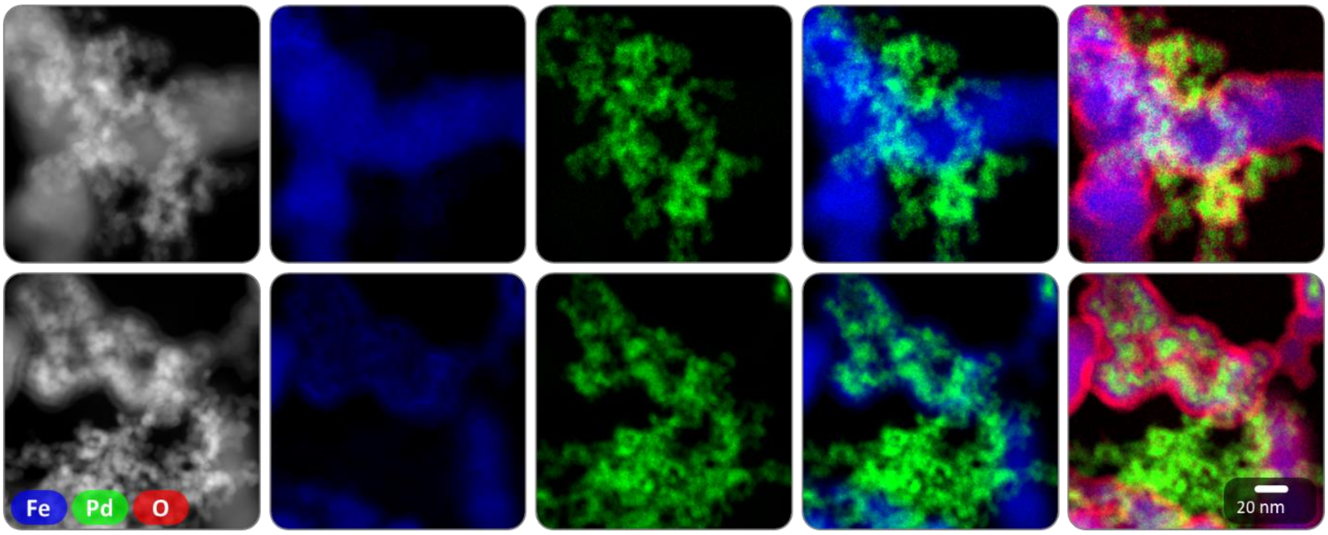

Figure S25 STEM-EDX-based element mapping of Pd@Fe gels synthesized under standard mortar conditions without magnetic field and an aqueous Pd suspension as substitute for H_2O. Both Fe and Pd form individual networks of different dimensions and ligament size, whereby Fe additionally acts as carrier for the Pd gel fragments.

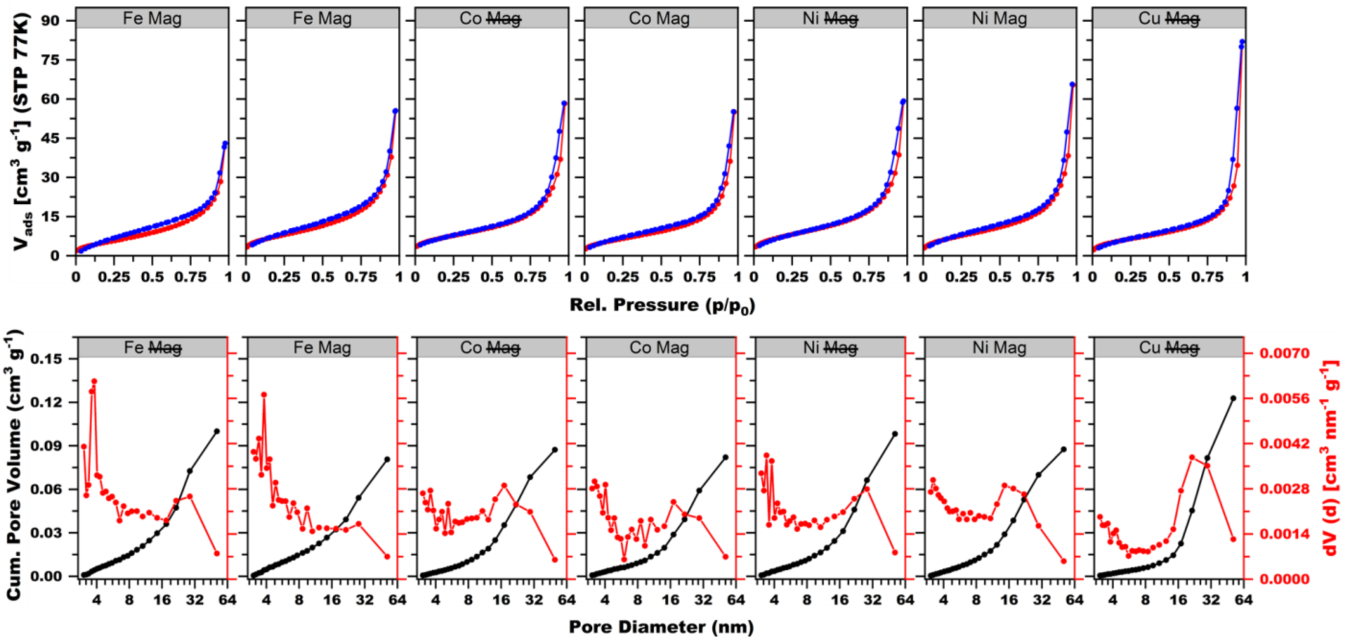

Figure S26 Nitrogen physisorption of the Fe, Co, Ni, and Cu base metal aerogels synthesized under up-scaled mortar conditions with and without applying an external magnetic field before adding water.

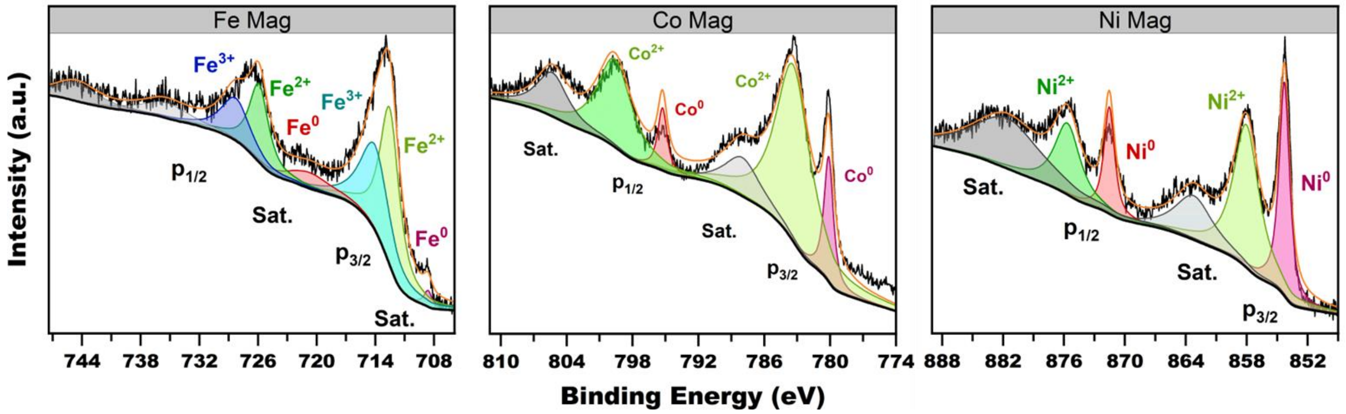

Figure S27 XPS spectra of the Fe, Co, and Ni base metal aerogels synthesized under up-scaled mortar conditions and applying an external magnetic field before adding water. The surface composition comprises of a metallic and oxidic phases. The amount of the latter decreases in the order Fe, Co, Ni.

References

(1) Bigall, N. C.; Herrmann, A. K.; Vogel, M.; Rose, M.; Simon, P.; Carrillo-Cabrera, W.; Dorfs, D.; Kaskel, S.; Gaponik, N.; Eychmüller, A. Hydrogels and Aerogels from Noble Metal Nanoparticles. *Angewandte Chemie - International Edition* **2009**, *48*, 9731–9734.

(2) Qian, F.; Troksa, A.; Fears, T. M.; Nielsen, M. H.; Nelson, A. J.; Baumann, T. F.; Kucheyev, S. O.; Han, T. Y. J.; Bagge-Hansen, M. Gold Aerogel Monoliths with Tunable Ultralow Densities. *Nano Lett* **2020**, *20*, 131–135.

(3) Henning, S.; Kühn, L.; Herranz, J.; Durst, J.; Binninger, T.; Nachtegaal, M.; Werheid, M.; Liu, W.; Adam, M.; Kaskel, S.; Eychmüller, A.; Schmidt, T. J. Pt-Ni Aerogels as Unsupported Electrocatalysts for the Oxygen Reduction Reaction. *J Electrochem Soc* **2016**, *163*, F998–F1003.

(4) Fikry, M.; Weiß, N.; Bozzetti, M.; Ünsal, S.; Georgi, M.; Khavlyuk, P.; Herranz, J.; Tileli, V.; Eychmüller, A.; Schmidt, T. J. Up-Scaled Preparation of Pt-Ni Aerogel Catalyst Layers for Polymer Electrolyte Fuel Cell Cathodes. *ACS Appl Energy Mater* **2024**, *7*, 896–905.

(5) Feng, J. J.; Lin, X. X.; Chen, L. X.; Liu, M. T.; Yuan, J.; Wang, A. J. Ionic Liquid-Assisted Synthesis of Composition-Tunable Cross-Linked AgPt Aerogels with Enhanced Electrocatalysis. *J Colloid Interface Sci* **2017**, *498*, 22–30.

(6) Tang, S.; Vongehr, S.; Wang, Y.; Cui, J.; Wang, X.; Meng, X. Versatile Synthesis of High Surface Area Multi-Metallic Nanosponges Allowing Control over Nanostructure and Alloying for Catalysis and Sers Detection. *J Mater Chem A Mater* **2014**, *2*, 3648–3660.

(7) Liu, R.; Liu, J. F.; Jiang, G. Bin. Use of Triton X-114 as a Weak Capping Agent for One-Pot Aqueous Phase Synthesis of Ultrathin Noble Metal Nanowires and a Primary Study of Their Electrocatalytic Activity. *Chemical Communications* **2010**, *46*, 7010–7012.

(8) Yan, S.; Mahyoub, S. A.; Lin, J.; Zhang, C.; Hu, Q.; Chen, C.; Zhang, F.; Cheng, Z. Au Aerogel for Selective CO_2 Electroreduction to CO: Ultrafast Preparation with High Performance. *Nanotechnology* **2022**, *33*, 125705.

(9) Zhao, W.; Huang, D.; Yuan, Q.; Wang, X. Sub-2.0-Nm Ru and Composition-Tunable RuPt Nanowire Networks. *Nano Res* **2016**, *9* (10), 3066–3074.

(10) Qian, F.; Lan, P. C.; Freyman, M. C.; Chen, W.; Kou, T.; Olson, T. Y.; Zhu, C.; Worsley, M. A.; Duoss, E. B.; Spadaccini, C. M.; Baumann, T.; Han, T. Y. J. Ultralight Conductive Silver Nanowire Aerogels. *Nano Lett* **2017**, *17*, 7171–7176.

(11) Peng, F.; Zhu, W.; Fang, Y.; Fu, B.; Chen, H.; Ji, H.; Ma, X.; Hang, C.; Li, M. Ultralight and Highly Conductive Silver Nanowire Aerogels for High-Performance Electromagnetic Interference Shielding. *ACS Appl Mater Interfaces* **2023**, *15*, 4284–4293.

(12) Zhang, D.; Bu, J.; Dou, X.; Yan, Y.; Liu, Q.; Wang, X.; Sun, Z.; Guo, G.; Zheng, K.; Deng, J. Ultra-Large Two-Dimensional Metal Nanowire Networks by Microfluidic Laminar Flow Synthesis for Formic Acid Electrooxidation. *Angewandte Chemie - International Edition* **2024**, *63*, e202408765.

(13) Liu, W.; Rodriguez, P.; Borchardt, L.; Foelske, A.; Yuan, J.; Herrmann, A. K.; Geiger, D.; Zheng, Z.; Kaskel, S.; Gaponik, N.; Kötz, R.; Schmidt, T. J.; Eychmüller, A. Bimetallic Aerogels: High-Performance Electrocatalysts for the Oxygen Reduction Reaction. *Angewandte Chemie - International Edition* **2013**, *52*, 9849–9852.

(14) Burpo, F. J.; Nagelli, E. A.; Morris, L. A.; McClure, J. P.; Ryu, M. Y.; Palmer, J. L. Direct Solution-Based Reduction Synthesis of Au, Pd, and Pt Aerogels. *J Mater Res* **2017**, *32*, 4153–4165.

(15) Georgi, M.; Klemmed, B.; Benad, A.; Eychmüller, A. A Versatile Ethanolic Approach to Metal Aerogels (Pt, Pd, Au, Ag, Cu and Co). *Mater Chem Front* **2019**, *3*, 1586–1592.

(16) Zheng, J. N.; Zhang, M.; Li, F. F.; Li, S. S.; Wang, A. J.; Feng, J. J. Facile Synthesis of Pd Nanochains with Enhanced Electrocatalytic Performance for Formic Acid Oxidation. *Electrochim Acta* **2014**, *130*, 446–452.

(17) Wang, Y.; Lv, H.; Sun, L.; Guo, X.; Xu, D.; Liu, B. Ultrathin and Wavy PdB Alloy Nanowires with Controlled Surface Defects for Enhanced Ethanol Oxidation Electrocatalysis. *ACS Appl Mater Interfaces* **2021**, *13*, 17599–17607.

(18) Wang, Y.; Shi, Y. F.; Chen, Y. B.; Wu, L. M. Hydrazine Reduction of Metal Ions to Porous Submicro-Structures of Ag, Pd, Cu, Ni, and Bi. *J Solid State Chem* **2012**, *191*, 19–26.

(19) Zhao, H.; Yuan, Y.; Zhang, D.; Qin, Y.; Han, Y.; Li, H.; Wang, Z.; Li, S. X.; Lai, J.; Wang, L. Ultrafast Generation of Nanostructured Noble Metal Aerogels by a Microwave Method for Electrocatalytic Hydrogen Evolution and Ethanol Oxidation. *ACS Appl Nano Mater* **2021**, *4*, 11221–11230.

(20) Martínez-Lázaro, A.; Ramírez-Montoya, L. A.; Ledesma-García, J.; Montes-Morán, M. A.; Gurrola, M. P.; Menéndez, J. A.; Arenillas, A.; Arriaga, L. G. Facile Synthesis of Unsupported Pd Aerogel for High Performance Formic Acid Microfluidic Fuel Cell. *Materials* **2022**, *15*, 1422–1436.

(21) Ksar, F.; Surendran, G.; Ramos, L.; Keita, B.; Nadjo, L.; Prouzet, E.; Beaunier, P.; Hagège, A.; Audonnet, F.; Remita, H. Palladium Nanowires Synthesized in Hexagonal Mesophases: Application in Ethanol Electrooxidation. *Chemistry of Materials* **2009**, *21*, 1612–1617.

(22) Wang, Y.; Choi, S. Il; Zhao, X.; Xie, S.; Peng, H. C.; Chi, M.; Huang, C. Z.; Xia, Y. Polyol Synthesis of Ultrathin Pd Nanowires via Attachment-Based Growth and Their Enhanced Activity towards Formic Acid Oxidation. *Adv Funct Mater* **2014**, *24*, 131–139.

(23) Calabro, R. L.; Longstaff, G. L.; Tang, E. M.; Xiao, V. M.; Zammit, A. S.; Zhang, F. W.; Nagelli, E. A.; Chapman, P. H.; Lawton, T. J.; Allen, M. A.; Losch, A. R.; Palmer, J. L.; Ciampa, A. D.; Burpeau, I. Z.; Lucian, V. M.; Mandes, G. T.; Bartolucci, S. F.; Maurer, J. A.; Burpo, F. J. Magnetic-Field-Assisted Fe Nanowire Conformable Aerogels Galvanically Displaced to Cu and Pt for Three-Dimensional Electrode Applications. *ACS Appl Mater Interfaces* **2025**, *17* (18), 26854–26870.

(24) Li, R.; Gou, X.; Li, X.; Wang, H.; Ruan, H.; Xiong, Y.; Tang, X.; Li, Y.; Yang, P. A. Improved Stretchable and Sensitive Fe Nanowire-Based Strain Sensor by Optimizing Areal Density of Nanowire Network. *Molecules* **2022**, *27*, 4717.

(25) Krajewski, M.; Liou, S. C.; Chiou, W. A.; Tokarczyk, M.; Małolepszy, A.; Płocińska, M.; Witecka, A.; Lewińska, S.; Ślawska-Waniewska, A. Amorphous Fe_xCo_{1-x} Wire-like Nanostructures Manufactured through Surfactant-Free Magnetic-Field-Induced Synthesis. *Cryst Growth Des* **2020**, *20*, 3208–3216.

(26) Lin, W. S.; Jian, Z. J.; Lin, H. M.; Lai, L. C.; Chiou, W. A.; Hwu, Y. K.; Wu, S. H.; Chen, W. C.; Yao, Y. D. Synthesis and Characterization of Iron Nanowires. *Journal of the Chinese Chemical Society* **2013**, *60*, 85–91.

(27) Li, X.; Guo, X.; Liu, T.; Zheng, X.; Bai, J. Shape-Controlled Synthesis of Fe Nanostructures and Their Enhanced Microwave Absorption Properties at L-Band. *Mater Res Bull* **2014**, *59*, 137–141.

(28) Bian, E.; Xu, Y.; Lou, S.; Fu, Y.; Zhou, S. Fabrication High-Purity Fe Nanochains with near Theoretical Limit Value of Saturation Magnetization of Bulk Fe. *Journal of Nanoparticle Research* **2016**, *18*, 331.

(29) Liang, C.; Pan, W.; Zou, P.; Liu, P.; Liu, K.; Zhao, G.; Fan, H. J.; Yang, C. Highly Conductive and Mechanically Robust NiFe Alloy Aerogels: An Exceptionally Active and Durable Water Oxidation Catalyst. *Small* **2022**, *18*, 2203663–2203672.

(30) Yan, S.; Zhong, M.; Wang, C.; Lu, X. Amorphous Aerogel of Trimetallic FeCoNi Alloy for Highly Efficient Oxygen Evolution. *Chemical Engineering Journal* **2022**, *430*, 132955–132962.

(31) Zou, P.; Li, J.; Zhang, Y.; Liang, C.; Yang, C.; Fan, H. J. Magnetic-Field-Induced Rapid Synthesis of Defect-Enriched Ni-Co Nanowire Membrane as Highly Efficient Hydrogen Evolution Electrocatalyst. *Nano Energy* **2018**, *51*, 349–357.

(32) You, W.; Pei, K.; Yang, L.; Li, X.; Shi, X.; Yu, X.; Guo, H.; Che, R. In Situ Dynamics Response Mechanism of the Tunable Length-Diameter Ratio Nanochains for Excellent Microwave Absorber. *Nano Res* **2020**, *13*, 72–78.

(33) Huang, H.; Lai, F.; Fu, H.; Chen, Y.; Li, H.; He, F.; Wang, Z.; Zhang, N.; Bai, S.; Liu, T. Spin-Engineered Cu-Ni Metallic Aerogels for Enhanced Ethylamine Electrosynthesis from Acetonitrile. *J Mater Chem A Mater* **2023**, *11*, 2210–2217.

(34) Zhao, J.; Malgras, V.; Na, J.; Liang, R.; Cai, Y.; Kang, Y.; Alshehri, A. A.; Alzahrani, K. A.; Alghamdi, Y. G.; Asahi, T.; Zhang, D.; Jiang, B.; Li, H.; Yamauchi, Y. Magnetically Induced Synthesis of Mesoporous Amorphous CoB Nanochains for Efficient Selective Hydrogenation of Cinnamaldehyde to Cinnamyl Alcohol. *Chemical Engineering Journal* **2020**, *398*, 125564.

(35) Tang, Y.; Yeo, K. L.; Chen, Y.; Yap, L. W.; Xiong, W.; Cheng, W. Ultralow-Density Copper Nanowire Aerogel Monoliths with Tunable Mechanical and Electrical Properties. *J Mater Chem A Mater* **2013**, *1*, 6723–6726.
